# Supplementary material for: Community Participatory Approach to Design, Test, and Implement Interventions That Reduce Risk of Bat-Borne Disease Spillover: A Case Study from Cambodia
Source: Trop Med Infect Dis. 2025 Dec 27;11(1):7. doi: 10.3390/tropicalmed11010007 (PMC12846364; doi:10.3390/tropicalmed11010007)
Supplement: Supplementary file 1 [file tropicalmed-11-00007-s001.zip › File S1. Cambodia Activity 1.2.6.1 Research On Bat Ecology and Prevalence of Pathogens.pdf]

ACTIVITY 1.2.6.1: RESEARCH REPORT ON BAT ECOLOGY AND PREVALENCE OF PATHOGENS

An Interim Report on Bat Guano Farms in Kang Meas District from STOP Spillover Cambodia

September 2023

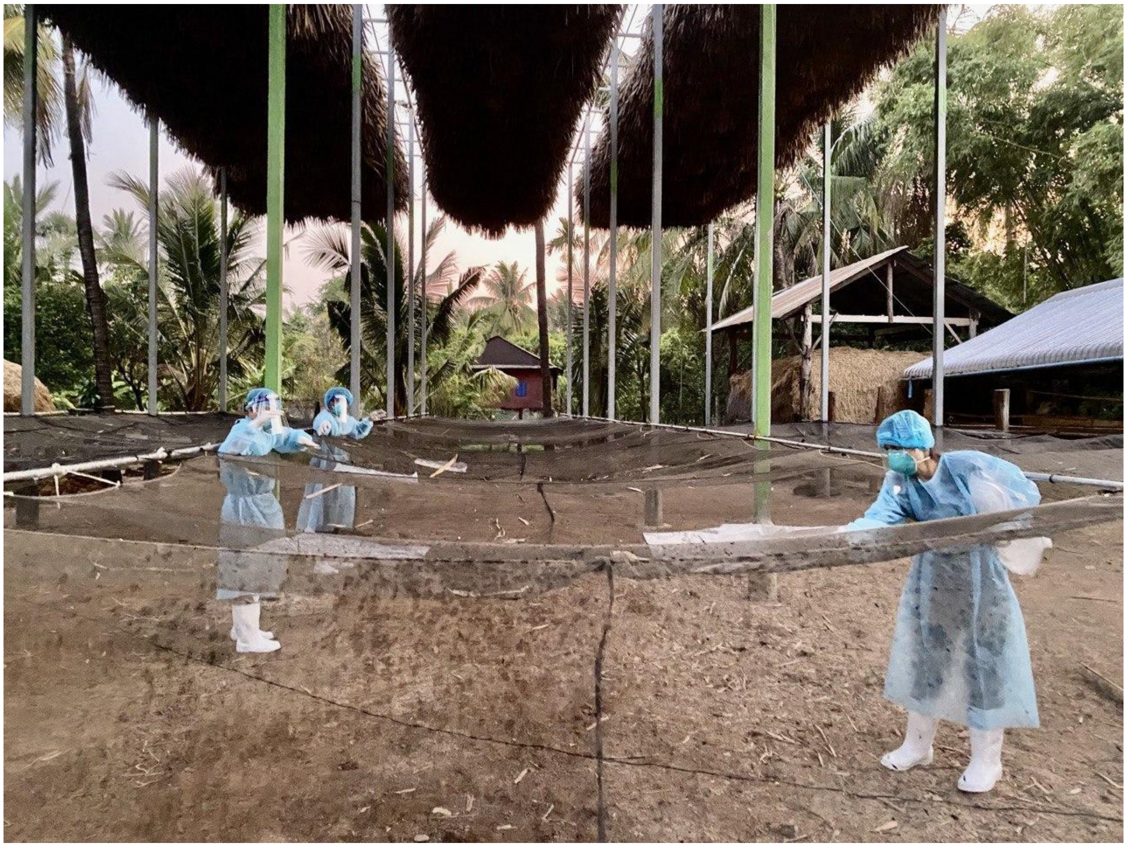

Cover image. The field team lays out plastic sheets for bat guano sample collection. Photo credit: STOP Spillover Cambodia.

CONTENTS

Executive Summary 4

Introduction 6

- Background 6
- Objectives 7

Methodology 8

- Site Selection 8
- Household Survey 8
- Bat Abundance Estimation 10
- Bat Species Identification 10
- Bat Guano Sampling 11
- Coronavirus PCR Testing 12
- Data Analysis 14

Results 15

- Demographics 15
- Roost Construction and History 15
- Guano Production 17
- Seasonal Patterns of Bat Abundance 18
- Bat Mortality 20
- Bat Species 20
- Bat Abundance Estimation 21
- Coronavirus Testing 22

Discussion 24

Literature cited 27

Appendices 29

STOP Spillover

Strategies to Prevent Spillover (or "STOP Spillover") enhances global understanding of the complex causes of the spread of a selected group of zoonotic viruses from animals to humans. The project builds government and stakeholder capacity in priority Asian and African countries to identify, assess, and monitor risks associated with these viruses and develop and introduce proven and novel risk reduction measures. In the context of this work "spillover" refers to an event in which an emerging zoonotic virus is transferred from a non-human animal host species (livestock or wildlife) to another or humans.

This report is made possible by the generous support of the American people through USAID. The contents are the responsibility of STOP Spillover and do not necessarily reflect the views of USAID or the United States Government.

List of Figures

Figure 1: Location of bat guano farms in Khchau commune, Kang Meas District, Kampong Cham province 9
Figure 2: Portraits of Cambodian bat species employed in interviews Image Credits: Neil Furey (images 1-10,12), Merlin Tuttle (image 11) 11
Figure 3: OH-DReaM Working Group members distribute the plastic sheets (left) and collect the bat guano and urine samples (right). Photo credit: STOP Spillover Cambodia........ 12
Figure 4: Examples of the different styles of bat roost. Traditional roost (left), linear roost (middle), and linear roost on artificial support (right). Photo credit: STOP Spillover Cambodia. 15
Figure 5: The self-reported number of bat roosts from 2017 to 2021. 16
Figure 6: Seasonal fluctuation in bat guano production. 18
Figure 7: Seasonal variation in farmer-reported relative bat abundance. 19
Figure 8: Spectrogram of search phase calls emitted by Scotophilus kuhlii in the study area...... 21
Figure 9: Installing the DVR camera system. Photo credit: STOP Spillover Cambodia. 21

List of Tables

Table 1: Primer sequences for PCR testing 13
Table 2: Roost area, number, and production by village from 16 bat guano farms. 17
Table 3: Example results from bat Emergence count at the first two bat guano farms 22
Table 4: Detections of coronaviruses in bat guano and urine samples (first sampling period) ... 22
Table 5: Initial phylogenetic analysis of coronavirus RNA found in pooled bat guano and urine samples (first sampling period) 23

List of Acronyms

CoVs	Coronaviruses
GHSA	Global Health Security Agenda
IPC	Institut Pasteur du Cambodge
JEE	Joint External Evaluation
OH-DReaM WG	One Health Design, Research and Mentorship Working Group

EXECUTIVE SUMMARY

In Cambodia, bat guano farming has been practiced for many years, but there is little formal scientific documentation of the farms or the practices of farmers. It has been identified that these farms possibly represent a high-risk interface for zoonotic spillover, but there is inadequate scientific knowledge to assess the real level of risk. Coronaviruses (CoVs) have been detected in bats worldwide but seasonal patterns of CoV shedding in bats in Asia are complex and not well understood. Alphacoronaviruses, which are generally believed to have low pathogenicity in humans, have been reported at guano farms, but to date, neither beta coronaviruses nor any other coronavirus known to have significance to human health has been found. However, only limited sampling has occurred, and uncertainty remains as to whether viruses with pandemic potential could occur at this interface and whether seasonal patterns of shedding exist, which may impact the effectiveness of surveillance.

This interim report describes progress in STOP Spillover Cambodia's study of bat guano farms in Kampong Cham province (Activity 1.2.6.1). The overall objective of this study is to (i) assess the scale and distribution of bat guano farming in Kang Meas District; (ii) identify the bat species and zoonotic pathogens present at bat guano farms; and (iii) evaluate the frequency and seasonality of CoV shedding by bats at bat guano farms. The data from this study will inform the design of interventions that reduce the risk of human exposure to bat-associated pathogens.

To address these objectives, we conducted: (i) a household survey of all bat guano farm owners in the target area focused on general information regarding bat roost ownership and scale of bat guano production; (ii) installation of a DVR camera system to quantify the number of bats emerging from selected bat roosts; (iii) acoustic monitoring combined with morphological identification of bat carcasses and interviews with bat farmers to identify the bat species present; (iv) collection of bat urine and fecal samples at bat guano-producing households; and (v) testing of samples for the presence of CoV RNAs to quantitatively assess viral spillover risk from bats.

Over the course of eight days of sampling in April-May 2023, 16 bat guano producing households were visited and interviewed, 256 samples were collected and sent to the laboratory of Institut Pasteur du Cambodge (IPC) to be tested for CoV RNA. A second guano sampling trip was conducted in August 2023 and two further trips are planned for project Year 4.

All farms used bunches of sugar palm leaves as roost material suspended either from palm trees or from man-made structures of timber, steel and/or concrete. Farms had an average of 124 m^2 of roost area and reported an average of 12-47 kg guano production per week depending on the

season. A distinct seasonal pattern in both respondent-observed bat abundance and guano production was apparent, which aligned with typical weather patterns in Cambodia. The wet season (June to December) represents the time when most farmers reported the highest bat abundance and guano production, whereas the lowest abundance and production estimates occurred during the dry season (January to May).

Respondents showed a high level of interest in and understanding of the bats on their farms. Over 80% could correctly identify the dominant species of bat on guano farms (*Scotophilus kuhlii*) from a lineup of anonymous images, and respondents could generally predict accurately the time and direction of emergence of the bats.

Preliminary lab testing results returned a higher proportion of positive results from fecal samples than urine. Only pooled testing is reported at this stage, but all positive detections at this stage were ⩾ 98% identical to known alphacoronaviruses associated with bats in southeast Asia. None of the closest matches were betacoronaviruses or known human/zoonotic pathogens. In-depth phylogenetic analysis as well as examination of seasonal patterns of shedding will only be possible once data collection is complete. Field and lab work on this activity will continue in project Year 4. The end of fieldwork is anticipated in February or March 2024 with lab work also scheduled for completion within project Year 4.

INTRODUCTION

BACKGROUND

Infectious diseases remain an important threat to global health, as demonstrated by the recent emergence of SARS-CoV-2 (Zhou, 2020). Within a matter of months, the COVID-19 pandemic led to widespread restrictions on movement and commerce, affecting billions of people around the world, with long-term political and economic impacts ongoing and difficult to estimate (Wang. et al, 2020). At the time of this writing the death toll is estimated to be close to six million (WHO, 2023). Coronaviruses (CoVs) are classified into four genera: *Alphacoronavirus*, *Betacoronavirus*, *Gammacoronavirus*, and *Deltacoronavirus*. In humans, only viruses in the genera *Alphacoronavirus* and *Betacoronavirus* have been found to cause disease (Wong et al, 2019). CoVs can infect a wide yearneurological systems (Wong et al, 2019) and have been detected worldwide, but only the *Alphacoronavirus* and *Betacoronavirus* genera have been reported in bats (Woo, 2012). Phylogenetic studies suggest that progenitors of SARS-CoV-2 could have originated in rhinolophid bats in Southeast Asia (Anderson, 2020; Zhou, 2020). Bat reproductive phenology is linked to seasonal climatic conditions, and this has been identified as an important factor influencing circulation of some pathogens including filoviruses (Amman et al., 2012) and paramyxoviruses (USAID PREDICT, 2020). Seasonal patterns of CoV shedding in bats in Asia are complex and not well-defined, though some data point to an association with reproductive phenology, which is, in turn, climate-driven (Marinda et al., 2021).

Bat guano has been harvested in Cambodia for many years and the construction of artificial roosts to attract insectivorous bats and collect their guano for use as fertilizer has increased in rural communities in Cambodia over recent years (Chhay, 2012). Seventeen viruses including 16 novel viruses (4 coronaviruses, 4 paramyxoviruses and 8 rhabdoviruses) and one previously known coronavirus have been reported by USAID PREDICT from bats in Cambodia (PREDICT Cambodia, 2020). These data indicate that some alphacoronaviruses were found on the two farms where bats were sampled during the project, but these were not classified as highly likely to be pathogenic in humans based on phylogenetic analysis (PREDICT, unpubl. data). Only a small amount of sampling was conducted during the PREDICT project and although some additional sampling was undertaken by the CamBatRat project, no further results of testing from the bat guano farms of Kampong Cham province since 2018 are available (Duong Veasna, Pers. Comm.). As such, it remains uncertain whether viruses with pandemic potential can be found in these farms, and what the seasonal patterns of shedding may be.

Previous records have only documented *Scotophilus kuhlii* (Lesser asiatic yellow house bat) in the guano farms, but it is not known whether other species may be present at some farms as many have not previously been surveyed. Knowledge gaps also remain regarding the number and scale of bat guano farms within Kampong Cham province and nationally.

In this study, we conducted a comprehensive survey of bat guano farms in the Kang Meas district of Kampong Cham province to document their number, location and scale. Sampling of bat urine and guano from the farms was also conducted to investigate the presence of CoVs and strain diversity. This activity will contribute to zoonotic disease surveillance in the Global Health Security Agenda (GHSA) (P5.1) and risk communication (R5.1). In addition, the research will help strengthen Cambodia's Joint External Evaluation (JEE) scores related to zoonotic disease surveillance, coordination and private sector engagement in future evaluations.

OBJECTIVES

This study aimed to use information from the laboratory testing and household surveys to develop interventions to reduce the risk of virus spillover from bat guano farms within the study region. Key objectives were as follows:

- To assess the scale and distribution of bat guano farming in Kang Meas District, Kampong Cham Province
- To identify the bat species and the coronaviruses present at bat guano farms
- To evaluate the frequency and seasonality of coronavirus shedding by bats at bat guano farms
- To provide scientific data to inform the design of interventions that reduce the risk of exposure to bat-associated pathogens

METHODOLOGY

SITE SELECTION

The study area (Figure 1) was selected following a national stakeholder engagement meeting which discussed the status of spillover risks in Cambodia and prioritized the interfaces, pathogens, and geographic locations for STOP Spillover interventions in Cambodia. The bat-human interface selected for the present study was a bat guano producing community located in the central lowlands of the Mekong River in south-eastern Cambodia. This area is inundated when water levels in the Mekong River rise during the wet season.

HOUSEHOLD SURVEY

Interviews were conducted at 16 bat guano producing households by five members of the One Health Design, Research and Mentorship Working Group (OH-DReaM WG) who were trained, oriented and supervised for the purposes of data collection. The OH-DReaM WG members visited bat guano farms and conducted a 30-minute questionnaire. The questionnaire focused on general information regarding bat roost ownership and locality; scale of production, roost construction, history and quantity of bat guano harvested; the bat species, volume of bat roost, and fluctuation of bat populations over time. Data were entered using KoboToolbox (Kobo, Cambridge, MA, USA) on a tablet. All survey data collection was completed in April and May 2023.

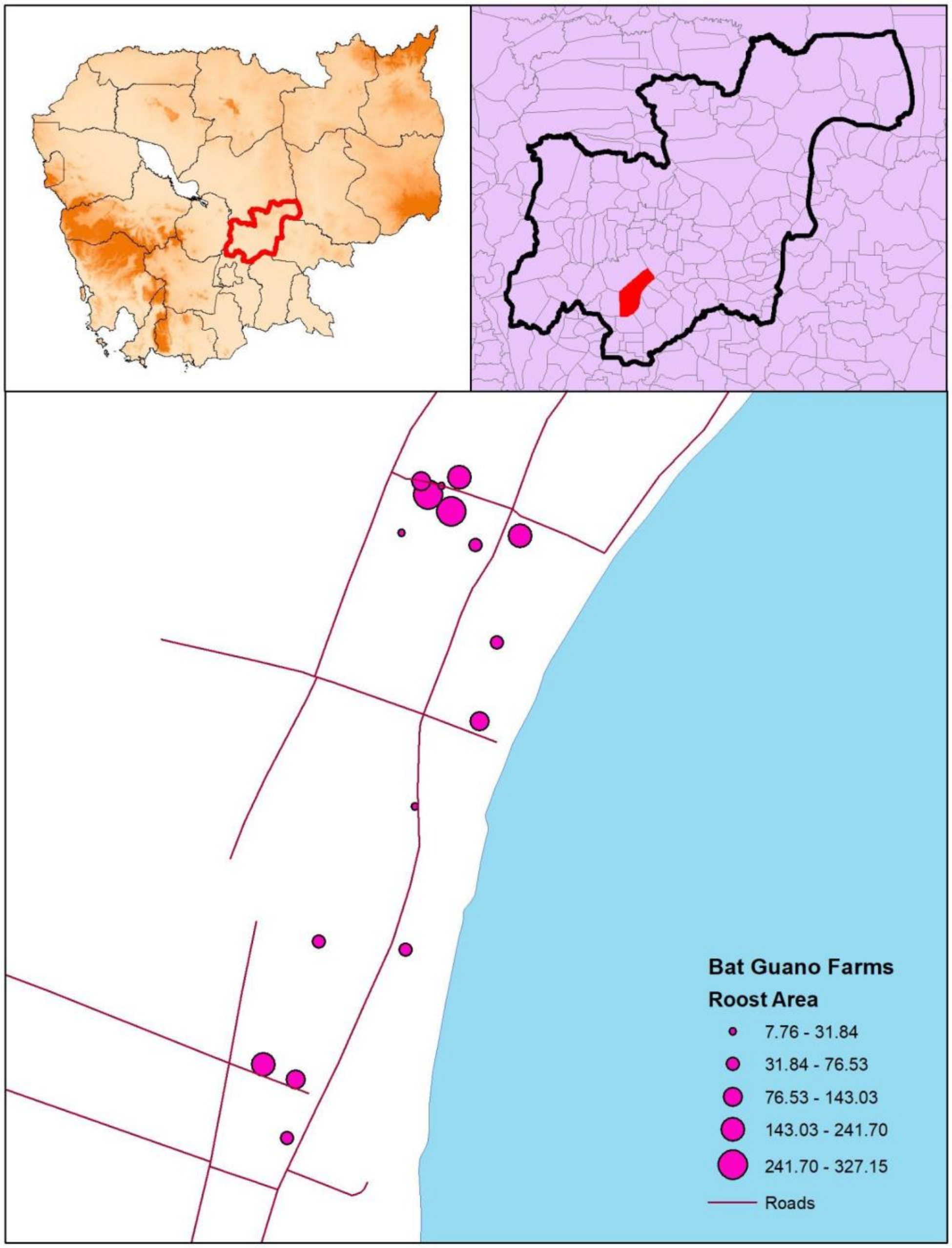

Figure 1: Location of bat guano farms in Khchau commune, Kang Meas District, Kampong Cham province

BAT ABUNDANCE ESTIMATION

To estimate the number of bats present at guano farms, a wired DVR camera system comprising four infrared-supplemented video cameras, a DVR recorder, and a 12V battery was installed at four farms for one night each. We targeted the evening emergence period when bats leave the roost to feed. Roost populations were initially quantified by:

- Counting the total number of departing bats recorded by the four cameras for one hour, starting from the time of first departing bat.
- Recording the time of first and last departures of bats (as well as any returns) during the first hour for each camera to assess whether a one-hour count was genuinely sufficient and avoiding possible double-counts due to returning bats.
- Ranking the likely accuracy and level of confidence for each count based on camera framing and bat visibility.

BAT SPECIES IDENTIFICATION

During farm visits, opportunistic searches were undertaken for bat carcasses underneath roost structures. These were photographed, measured for forearm length and identified in accordance with standard morphological criteria (Kruskop, 2013; Francis, 2019). Additionally, during the interviews, the owner or operator of each bat guano farm was presented with 12 unlabeled (anonymous) images of 11 different bat species known to occur in Cambodia (Figure 2) and asked to select any species that they believed to be present on their farms.

Lastly, acoustic data were collected at each guano farm using Song Meter 4 full-spectrum bat detectors fitted with calibrated U2 ultrasound microphones (Wildlife Acoustics, Maynard MA, USA). A single device was deployed overnight for one night at each farm and programmed to record from 30 minutes before sunset until sunrise. These data were retained for analysis in case the opportunistic carcass searches and species interviews failed to provide adequate confidence regarding the bat species present at each guano farm.

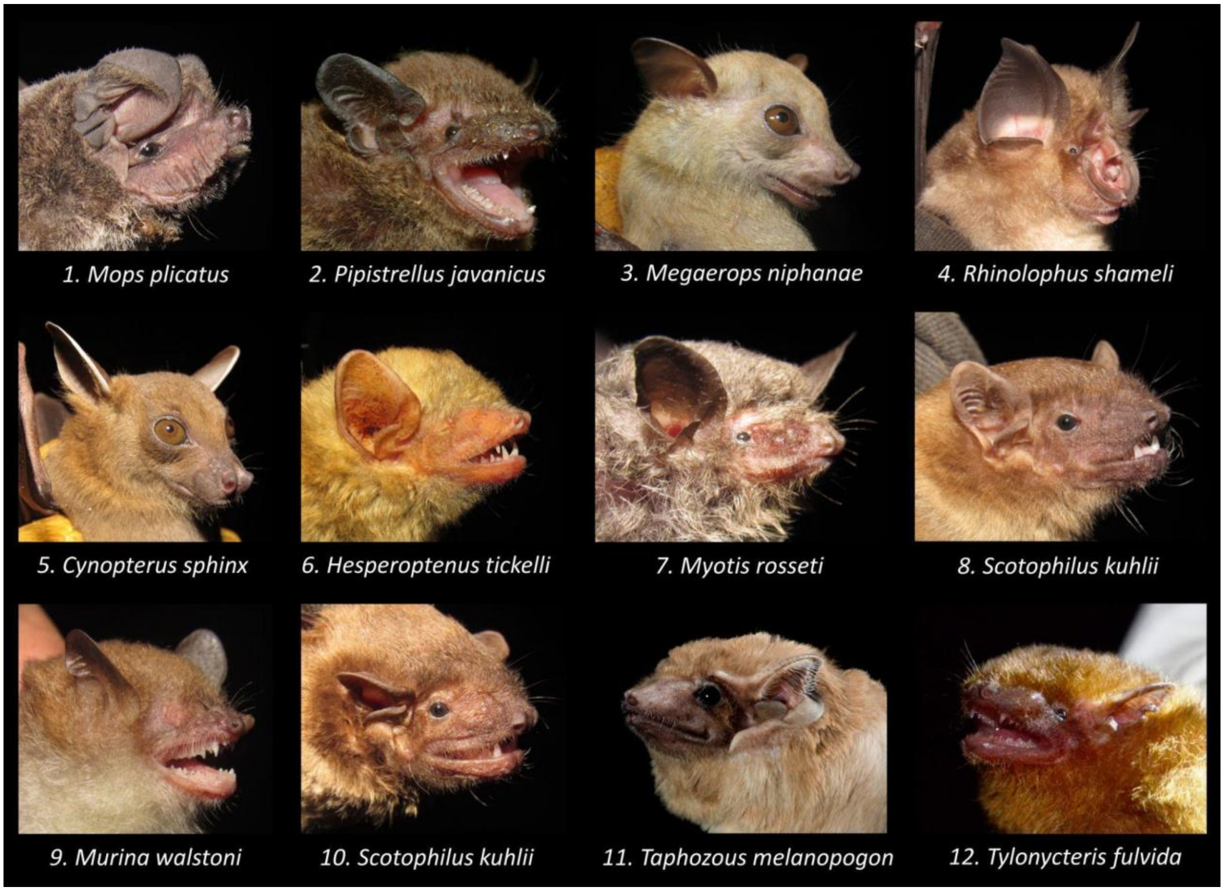

Figure 2: Portraits of Cambodian bat species employed in interviews. Image Credits: Neil Furey (images 1-10,12), Merlin Tuttle (image 11).

BAT GUANO SAMPLING

Guano and urine samples were collected non-invasively by placing plastic sheets underneath multiple roosts. Approximately 1 square meter of plastic sheet was used per ten square meters of occupied roosting area. After 15 minutes, fecal material captured by the plastic sheet was collected using plastic straws, while the urine was collected by sterile polyester swabs (Copan Diagnostics, Murrieta CA, USA). The guano samples and pooled urine samples were placed in a 2ml cryovial (CryoKING, Biologix group ltd, Changzhou, China), containing 0.9ml of DNA/RNAshield (Zymo Research, Irvine CA, USA) and kept on ice until reaching the laboratory, where they were kept frozen at <-20°C until testing. Personal protective equipment (PPE) and other biosafety procedures were in accordance with the STOP Spillover Biorisk Management Guide.

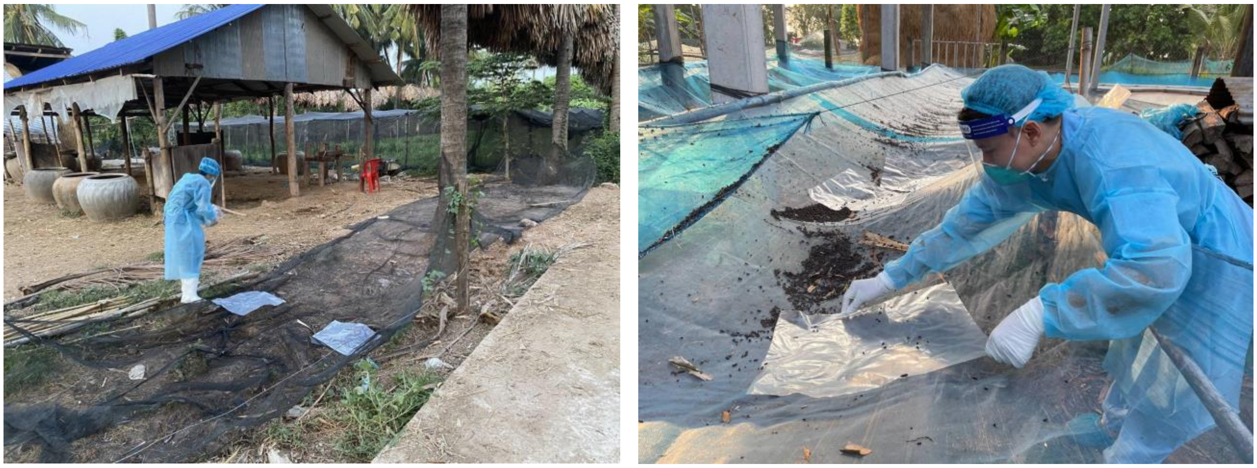

Figure 3: OH-DReaM Working Group members distribute the plastic sheets (left) and collect the bat guano and urine samples (right). Photo credit: STOP Spillover Cambodia.

CORONAVIRUS PCR TESTING

All samples were tested for presence of CoV RNA at the virology laboratory, Institut Pasteur du Cambodge (IPC). All samples were first centrifuged to remove any sample material from the caps before being homogenized using a pellet pestle. Subsequently, the samples were vortexed ~20 seconds and briefly centrifuged again. Afterward, the samples were pooled, with 5 samples per pool/100μl per individual. The pooled samples were then vortexed and centrifuged again (3000rpm for 15 minutes). The supernatant was recovered and filtered using a syringe filter with pore size of 0.45μl (Thermo Scientific™ Nalgene™ Sterile Syringe Filters).

RNA was extracted from each pool using the Zymo Research Direct-zol RNA MiniPrep kit (Zymo Research, CA, USA) and cDNA was transcribed using SuperScript III First-Strand Synthesis Super-Mix (Invitrogen, San Diego, CA) following the manufacturer's instructions. pan-CoV conventional hemi-nested RT-PCR targeting the RdRp gene was performed as previously described (Quan et al., 2010) in a 50μL reaction volume. Additionally, RNA samples were screened using an adapted duplex one-step real-time RT-PCR targeting the E and N genes of Sarbecovirus (Cornman et al., 2019). A 25μl reaction mixture was prepared using Superscript III one-step RT-PCR system with Platinum Taq Polymerase (Invitrogen, Darmstadt, Germany), consisting of 5μl of RNA, 12.5μl of 2X reaction buffer, 0.5μl of 50mM MgSO4 solution (Invitrogen), and 1μg of bovine serum albumin (Roche). Primers and probes were added at final

concentrations of 400nM and 200nM, respectively. The thermal cycling for the PCR amplification was carried out using the CFX96™ Real-Time PCR detection system (Bio-Rad). The cycling program consisted of an initial incubation at 55°C for 10 minutes, followed by a denaturation step at 94°C for 3 minutes. This was then followed by 45 cycles of denaturation at 94°C for 15 seconds and annealing/extension at 58°C for 30 seconds.

All positive samples by pan-CoV conventional RT-PCR were subsequently sent for Sanger sequencing at Macrogen, Inc. (Seoul, Republic of Korea). The sequencing was performed in both forward and reverse directions using the primers from the second round of the hemi-nested PCR. The sequences obtained were confirmed for similarity using the NCBI BLAST search (https://blast.ncbi.nlm.nih.gov/Blast.cgi). A sample was considered positive for CoV if any of the PCR systems used yielded a confirmed positive result.

Positive pools will be disaggregated and samples will be run individually to identify positive ones.

Table 1: Primer sequences for PCR testing.

	**Primer Sequence**	**Gene targeted**	**Ref**
**Real time RT-PCR**			
E_Sarbeco_F1	5'-ACAGGTACGTTAATAGTTAATAGCGT -3'	E	(Cornman VM, et al., 2019)
E_Sarbeco_R2	5'-ATATTGCAGCAGTACGCACACA-3'		
E_Sarbeco_P1	5'-FAM-ACACTAGCCATCCTTACTGCGCTTCG- BBQ-3'		
HKU-NF	5'-TAATCAGACAAGGAACTGATTA-3'	N	
HKU-NR	5'-CGAAGGTGTGACTTCCATG-3'		
HKU-NP	5'-FAM-GCAAATTGTGCAATTTGCGG-TAMRA-3'		
**Pan-CoV conventional RT-PCR**			
CoV-FWD1	CGTTGGIACWAAYBTVCCWYTICARBTRGG	RDRP	(Quan PL, et al., 2020)
CoV-RVS1	GGTCATKATAGCRTCAVMASWWGCNACATG		
CoV-FWD2	GGCWCCWCCHGGNGARCAATT		
CoV-RVS2	GGWAWCCCCAYTGYTGWAYRTC		

DATA ANALYSIS

Basic descriptive analyses, from the household survey, were performed in Excel (Microsoft 365). Other statistical analyses were performed using STATA/SE 10.1. All cartography and spatial analyses were performed using ArcGIS (ESRI, Redlands, CA, USA).

Mixed effects linear regression analysis was used to model the relationship between bat guano production and farm size with the addition of a random effect to account for correlation within villages.

Sequences were analyzed using Geneious Prime 2022.1.1 (Biomatters Ltd, Auckland, NZ) and Blast Search (NCBI). Nearest 100 matches were recovered from Genbank (NCBI) and pairwise identity used for comparison.

RESULTS

DEMOGRAPHICS

In total, 16 bat guano farm owners were interviewed with assistance from local authorities. The interviews took place in three villages within Khchau commune: Varint 1, Varint 2 and Varint 3. All participants were private individuals who operated their farms for daily subsistence and small-scale commerce.

ROOST CONSTRUCTION AND HISTORY

Though some farmers report that bat guano harvesting in Cambodia dates to the French colonial period (pre-1954) the interviewees started their farms more recently. Among the 16 interviewed farmers, two reported collecting bat guano in the Sangkom (post-colonial) period (1953-1970), whereas seven constructed their roosts in the post-Khmer Rouge period (1979-1991), five constructed their roosts in recent decades (1992-2010) and two more established their farms since 2020.

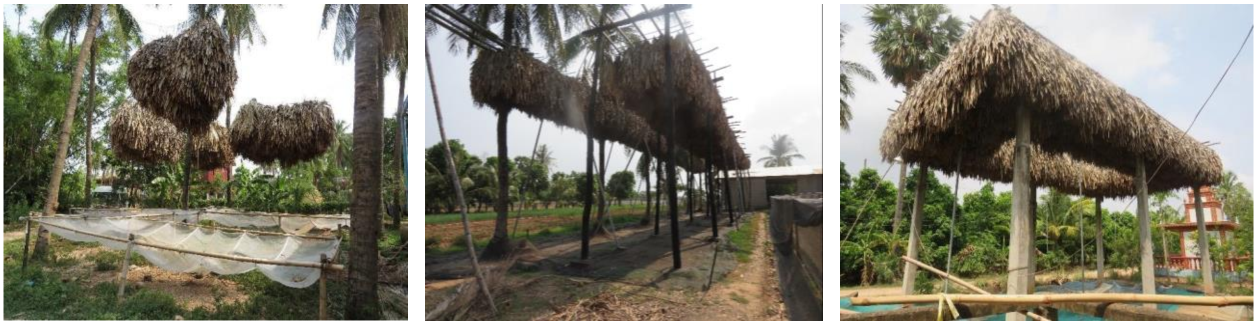

Figure 4: Examples of the different styles of bat roost. Traditional roost (left), linear roost (middle) and linear roost on artificial support (right). Photo credit: STOP Spillover Cambodia.

Three types of bat roost are employed in Kang Meas District. The simplest we term the “traditional roost,” which comprises additional bunches of sugar palm leaves attached to free-standing palm trees (Figure 4, left). To increase roost area, some farmers have strung additional wires or erected poles between trees (“linear roost with natural support”). Since about 2011, farmers report that they started to build the “linear roosts on artificial supports” and these now account for 10 of 16 (62%) of local guano farms. Of the remaining farms, three (19%) had linear roosts on natural supports and three had traditional dome roosts (19%).

All bat guano farms surveyed used a plastic netting to collect the guano, though some interviewees reported commencing this practice only recently. The stated purpose of the nets was to make cleaning easier and reduce losses to rain, though observations indicated it also reduced dust production and direct contact with guano while gathering it. Eleven farms raised the nets above ground level whereas the other five kept their nets at ground level.

All bat guano farmers used sugar palm leaf bunches as roosting material and reported changing half of the farm's bunches one year and the other half the following year. This is reportedly necessary to reduce parasite loads and thereby avoid bats abandoning the roosts. Since they began harvesting bat guano, 6 of the 16 farms (37.5%) reported they had increased the number of roosts whereas 10 of the 16 farms (62.5%) had not changed. Only 2 of the 16 farms (12.5%) had plans to increase their roosts next year whereas the rest planned to continue at the present scale.

There was no significant change in the number of roosts per farm over time (2017-2021), but the number of farms and hence total roost number increased (Figure 5).

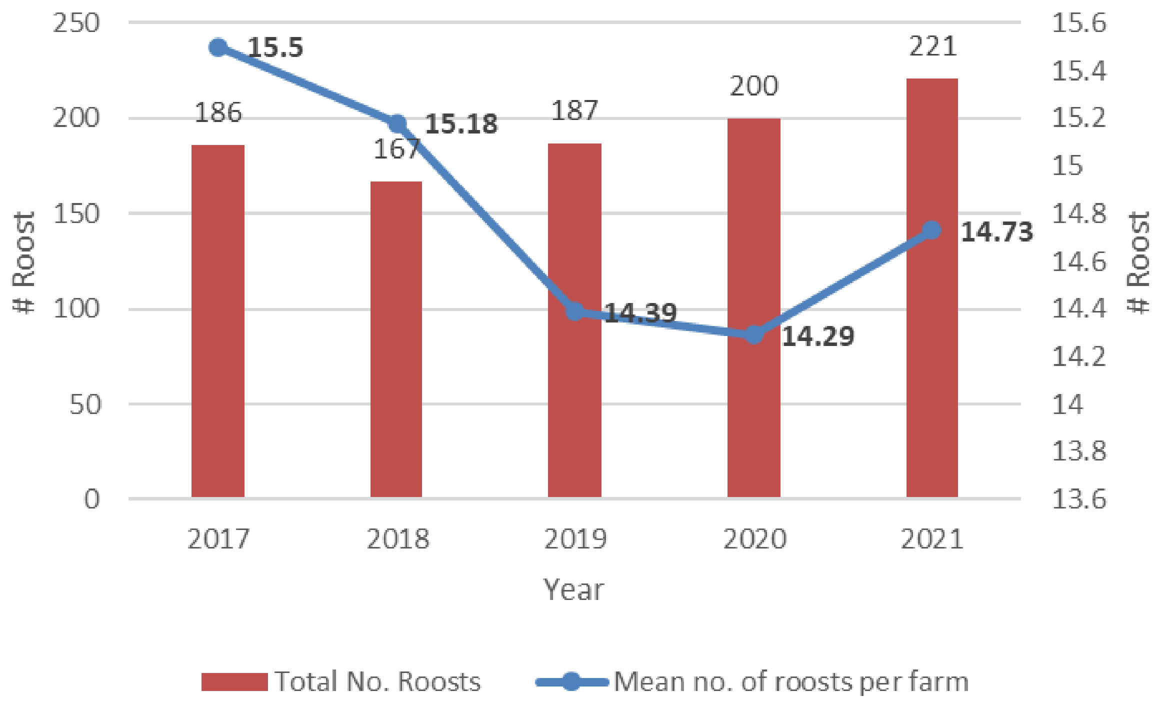

Figure 5: The self-reported number of bat roosts from 2017 to 2021.

GUANO PRODUCTION

The guano farms in the three villages (Table 2) were similar, but those in Varint 2 were smaller and produced somewhat less than those in the other two villages on average. The roost area was correlated with production.

Table 2: Roost area, number and production by village from 16 bat guano farms.

Village	N	Mean roost area (range) in m^2	Mean number of roosts (range)	Guano production (kg/week); mean (range)	
				Lowest period	Highest period
**Varint 1**	8	157.89 (14.3 - 327.15)	17.25 (5 - 44)	18.12 (6 - 42)	47 (13 - 175)
**Varint 2**	4	67.30 (7.76 - 143.03)	8.75 (1 - 17)	14.25 (5 - 25)	36 (10 - 87)
**Varint 3**	4	111.28 (49 - 217.64)	9.5 (3 - 22)	23.75 (4 - 49)	59.5 (20 - 120)

The roost area was significantly associated with bat guano production in the lowest period (Appendix 3), but this relationship was not significant regarding peak production in the highest producing period.

There was seasonal variation in the quantity of bat guano harvested throughout the year. A distinct seasonal pattern was apparent which aligned with typical weather patterns in Cambodia (Figure 6). The wet season (June to December) represents the time when most farmers reported the highest guano production, whereas the lowest producing period was reported during the dry season Jan to May).

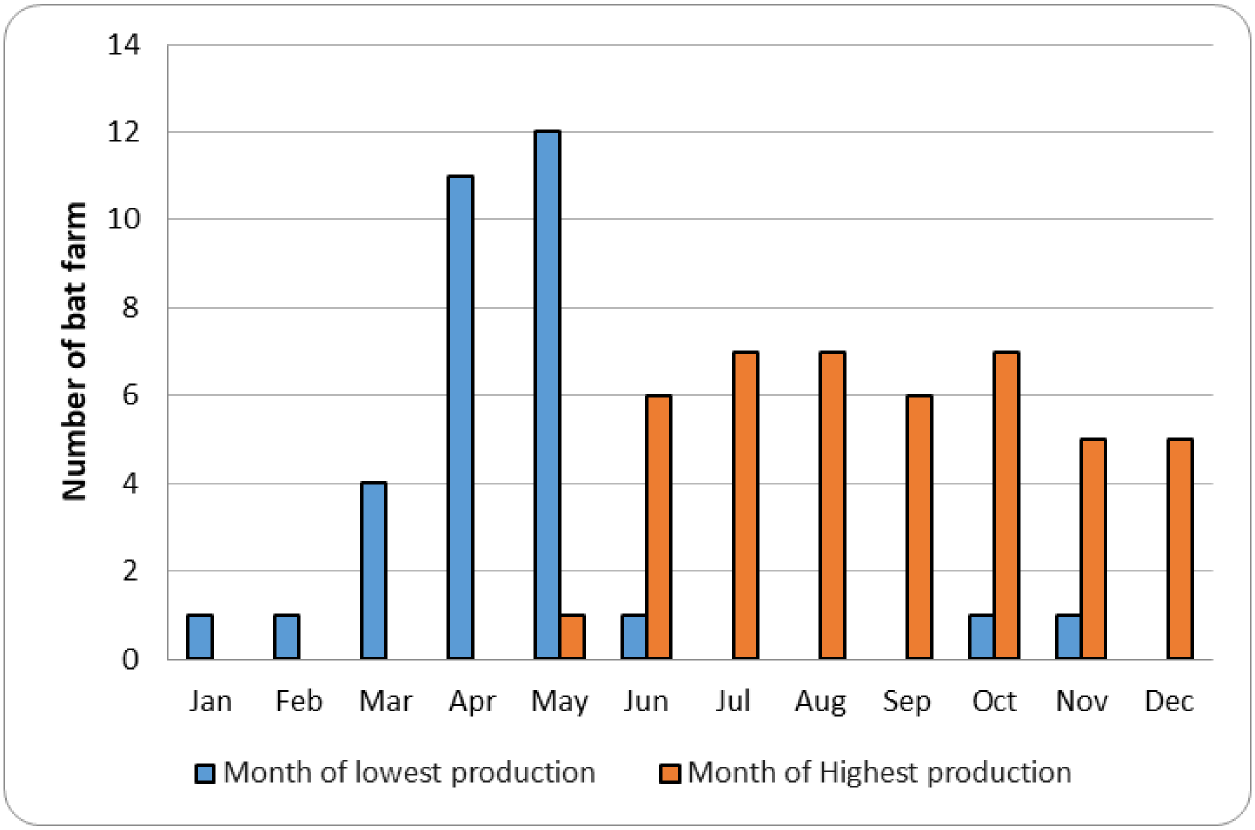

Figure 6: Seasonal fluctuation in bat guano production. Note: Most farmers reported a range of several months, so the sum of all observations is greater than the sample size.

SEASONAL PATTERNS OF BAT ABUNDANCE

All the study farms reported that numbers of bats vary throughout the year. The lowest estimated abundance was generally reported between February and May (dry season), while the highest reported abundance was typically in the rainy season from June to December (Figure 7).

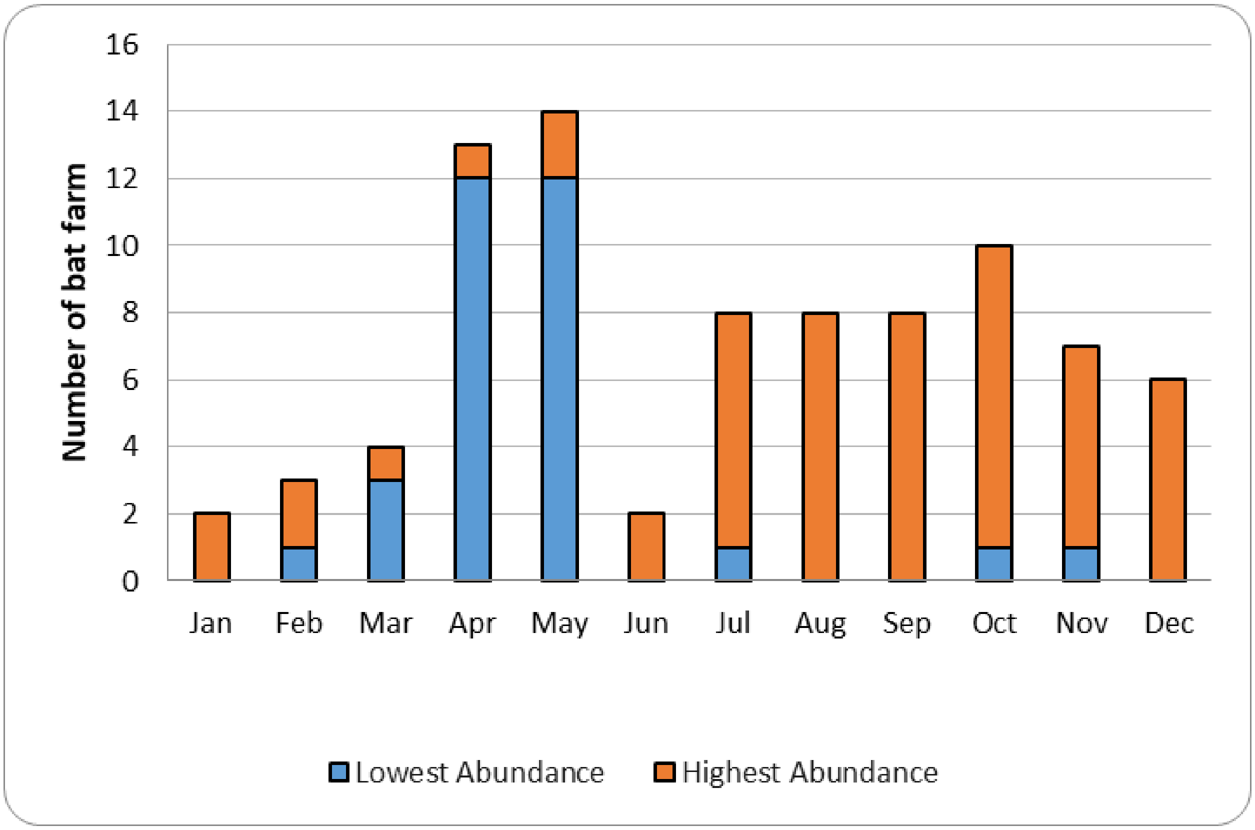

Figure 6: Seasonal variation in farmer-reported relative bat abundance.
Note: Most farmers reported a range of several months, so the sum of all observations is greater.

The respondents believed that seasonal low-point in abundance was caused by hot temperatures (40%) in the dry season (mostly in April-May), predators (33%) such as owls, lizards, and snakes disturbing the female bats during lactation, and the shortage of insects in the dry season (27%). Some respondents (19%) also felt that rain and strong wind suppressed numbers by keeping the bats in the roost without food. According to two farmers (13%), the bats will move from one roost to another if the palm leaves are old and have a bad smell.

Sixty-three percent of bat guano farmers (10 of 16) reported that the highest seasonal abundance of bats was caused by cooler weather which they believe promotes insect abundance while three (19%) felt that changing the roosting material out for new palm leaves was responsible. A further three farmers (19%) attributed the peak to the appearance of new juvenile bats. Only two of the 16 (13%) bat guano farmers thought that the rainfall contributes to causing the peak in abundance and one of 16 (6%) respondents reported the absence of predators (especially snakes) was behind this phenomenon.

Most (15 of 16) bat farmers reported observing the bats when harvesting guano, often when they had fallen from the roosts. Young bats were reported for two time periods – roughly December and April-May.

BAT MORTALITY

Fourteen farmers (87%) reported seeing more dead bats during heavy rain, strong wind, and times of highest and lowest temperatures. Some respondents said they see more dead bats in the dry season (9 of 16, 57%) in April-May and December to January while others said the rainy season (7 of 16, 44%) July-August. A variety of explanations (of varying biological plausibility and not all consistent with each other) were suggested by participants, including heavy rain, strong winds and low temperatures (9 of 16, 57%); high temperatures leading to reduced insect prey availability (7 of 16, 44%); bats falling from the roosts due to overcrowding or as young bats learn to fly (5 of 16, 32%). There is no scientific evidence to prove this claim yet.

Ten of 16 respondents (63%) reported having noticed an unusually large number of dead bats on certain occasions. For example, in July and August of both 2021 and 2022, an unusual mortality event was noticed by many farmers, who attributed the event to heavy rain keeping the bats in the roost without the opportunity to feed. Two of 16 farmers (13%) reported dead chickens and/or dogs at some time and they attributed these losses to hot temperatures, rather than the possibility of infections from bats.

BAT SPECIES

Among 15 respondents, 12 (80%) said that they knew the bat species on their farm. The local name for this bat is *prachheav angkam*. Based on the anonymous images of bat species shown to respondents, 12 (80%) identified an image of *Scotophilus kuhlii* (which is the dominant species present, based on prior studies and present work), whereas three (20%) selected an image of *Murina walstoni.*

Carcasses found opportunistically under roost structures were uniformly identified as *S. kuhlii,* though these have been found on only six farms to date (Farms 01, 04, 06, 09, 10, 14). Carcass collection will continue throughout the field work.

Acoustic recordings have been obtained at 14 farms to date and although other bat species naturally occur in the region, analyses of these recordings confirm the dominance of *S. kuhlii* at each farm, with all but a minority of signals closely matching those emitted by the species (Figure 8).

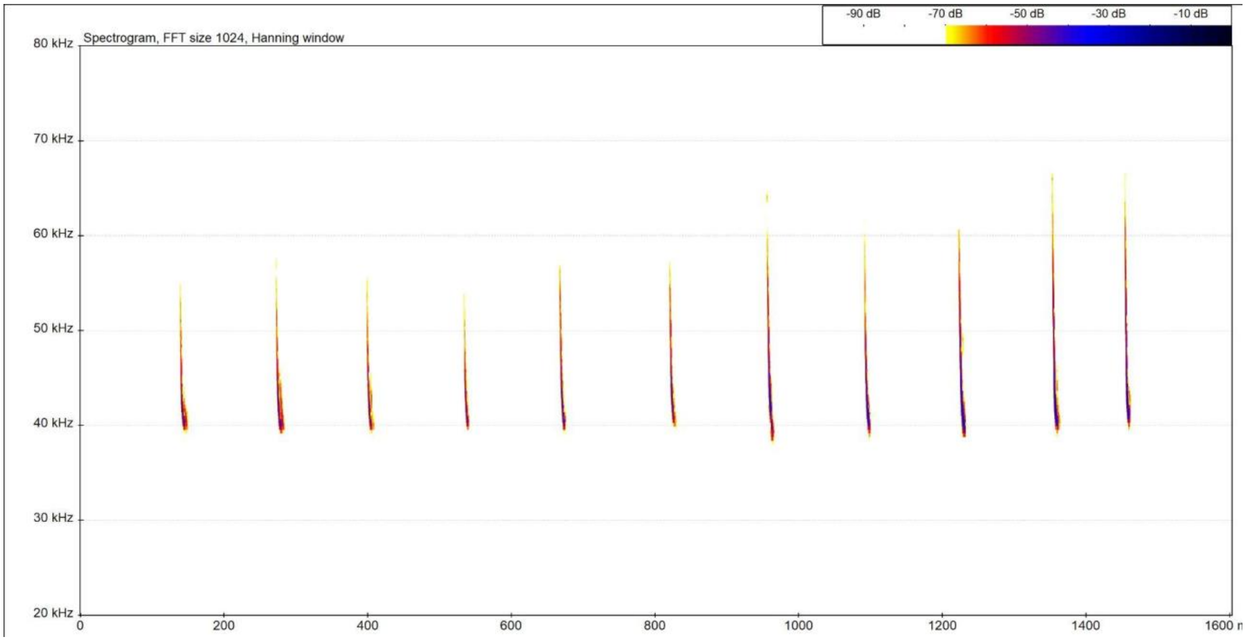

Figure 7: Spectrogram of search phase calls emitted by Scotophilus kuhlii in study area.

BAT ABUNDANCE ESTIMATION

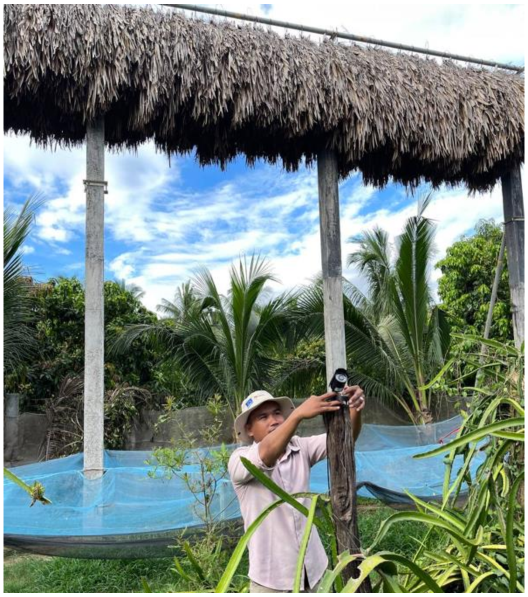

Figure 8: Installing the DVR camera system. Photo credit: STOP Spillover Cambodia.

To date, count data have been collected from five farms. Data for additional farms will be collected during the remaining two sampling periods with a total target of at least 10 farms. Usable video recordings were obtained from two of four attempts in April 2023 and only these have been analyzed to date (Table 5). Based on the initial results there are at minimum several thousand bats in the farms, though these counts will need to be adjusted for partial spatial coverage once data collection is complete. The time span of the emergence period seems to be tight, and this has been confirmed by our direct observations. Since the food availability (insect biomass) is at a nadir in April, a time when energetic demands are also high due to reproduction, it is assumed that most bats will emerge as early as possible each evening to forage for insects. The emergence period may not

be as compressed in the wet season when insect prey is likely more abundant. Data collection will continue during the remaining sampling trips targeting a minimum of 10 farms with usable data.

Table 3: Example results from bat Emergence count at the first two bat guano farms.

Farm ID	Date	First Hour Emergence Count		
		Time (1st Bat)	Time (Last Bat)	No. of bats counted
1	4/24/2023	18:07	18:28	**3340**
16	4/26/2023	17:41	18:27	**1909**

CORONAVIRUS TESTING

To detect CoVs present in bat guano, we collected and tested a total of 256 samples (141 fecal samples and 115 urine samples) in April and May 2023. Preliminary testing results (Table 4) showed that fecal samples returned a higher proportion of positive results than urine, though the exact proportion will not be known until the individual-level lab analysis is complete. All positive detections in the pooled stage were ⩾ 98% identical to known alphacoronaviruses associated with bats in southeast Asia. None of the closest matches were betacoronaviruses or known human/zoonotic pathogens.

Table 4: Detections of coronaviruses in bat guano and urine samples (first sampling period).

Sample Type	N		Results - First Sampling			
			Alpha-CoVs		Beta-CoV	
	Pools	Samples	Pos. Pools	Est. Pos*	Pos. Pools	Est. Pos*
Fecal	29	141	19	19.2%	0	0
Urine	24	115	12	12.9%	0	0

** Est. Pos = the expected value of the sample positivity rate based on the number of positive pools and the pool size. Individual-level data will be obtained when individual testing of positive pools is complete.*

Table 5: Initial phylogenetic analysis of coronavirus RNA found in pooled bat guano and urine samples (first sampling period).

Sample Matrix	GenBank Matches	% Pairwise Identity	Number of pools matching
Fecal	Alphacoronavirus sp. strain VZ_AlphaCoV_16715_47_c2, complete genome	⩾99%	8
	Alphacoronavirus sp. strain VZ_AlphaCoV_16715_61, complete genome	⩾98%	1
	Alphacoronavirus sp. strain VZ_AlphaCoV_16715_63, complete genome	⩾98%	3
	Alphacoronavirus sp. strain VZ_AlphaCoV_16715_7, complete genome	100%	3
	Alphacoronavirus sp. strain VZ_AlphaCoV_16715_77, complete genome	100%	3
	Alphacoronavirus sp. strain VZ_AlphaCoV_16715_86, complete genome	⩾98%	1
	Scotophilus bat coronavirus 512 2005/PREDICT_VN13F0043 RNA-dependent RNA polymerase mRNA, partial cds	⩾98%	1
Urine	Alphacoronavirus sp. strain VZ_AlphaCoV_16715_47_c2, complete genome	⩾99%	5
	Alphacoronavirus sp. strain VZ_AlphaCoV_16715_7, complete genome	100%	6
	Alphacoronavirus sp. strain VZ_AlphaCoV_16715_86, complete genome	⩾99%	1

DISCUSSION

Our study provides the first comprehensive survey of production and practices at bat guano farms and of the knowledge and beliefs of the bat guano farmers in Kang Meas District, Kampong Cham province. The roost area of farms was typically around 123.6m^2 and produced around 18.56kg of guano per week in the lowest period and 47.37kg of guano per week in the highest period. Data gathered in the risk reduction survey indicated that the guano sells for approximately $1.25/kg and most bat guano producers harvest two to three sacks of guano a week worth about $50 to $75 (20-22 kg per bag). Six bat guano farmers (out of 16 interviewed) reported that bat guano production represents their primary income source. There is a distinct seasonal pattern to guano production, with the highest production occurring in the wet season, which is to be expected as insect prey is most abundant during these months. The perceived number of bats on farms also correlates with this – farmers generally believed bat numbers in their roosts were at their lowest in the late dry season (March-May) and highest in June-December. This may be based simply on observed production, but it also reflects a plausible relationship to the breeding cycle, with pups generally born in April-May, so the population likely peaks at this time of year and this increase would become most noticeable once the juveniles begin to fly. It is unknown whether migration on and off the roosts plays any part in the observed seasonal variation in abundance.

Roost construction practices are evolving, and guano farms are becoming more popular. Farm sizes appear static, but the number of farms has increased. The use of nylon nets has increased and is now practiced by all farms in the three villages, although some only took this up recently, whereas others had done so for many years. Although traditional dome roosts still exist, there appears to be a gradual progression towards artificial support structures, culminating in the artificial modern roosts constructed entirely of steel, concrete and timber (plus the obligatory sugar palm leaves). According to data from the risk reduction survey, the key driver of this growth is the fact that guano farming is seen as an easy source of income – much more so than agriculture. Though some initial investment is needed, it becomes profitable with less labor than other enterprises.

In line with previous work, we have seen no evidence of other species of bats besides the lesser Asiatic yellow house bat (*Scotophilus kuhlii*) in bat guano farm roosts, although several other species are present in the area. A nearby temple is reported to have a substantial roost of *Taphozous spp.* in the roof of its dining hall. It appears that *S. kuhlii* is very adaptable to the artificial roost structures and the farms evidently provide attractive roosting habitat for the species. The guano farmers are highly aware of the bats on their farms and their movements. Farmers

accurately predicted the timing and direction of the emergence flights and most farmers (13 of 16) identified *S. kuhlii* from a lineup of 12 anonymous species images as the bat species inhabiting their farms. Only three selected another species - *Murina walstoni*. Although this species may appear similar to non-specialists, *M. walstoni* is highly unlikely to occupy roosts at any of the bat farms and certainly not in appreciable numbers because the species is naturally rare (sufficient for it to be considered Data Deficient by the IUCN [Csorba et al. 2020]), presently known only from singletons captured in very widely separated localities in mainland SE Asia (only 2-3 Cambodian records to date in remote areas of natural forest habitat), and, as with all members of the genus *Murina*, does not aggregate in large numbers (i.e., colonies are typically less than a dozen or so individuals).

Coronavirus RNA was found in 48.5% of pools with fecal samples returning a higher proportion of positive samples than urine (66% vs 50%). This leads to estimated individual-level positivity rates of 19.2% and 12.9% respectively. This is somewhat higher than previous work, though the difference between urine and feces is consistent with what has been seen in the past (Duong Veasna, pers. comm). Individual-level testing of all samples from positive pools is underway at the time of writing. Field work began in April 2023 and a second sampling trip (of four total planned) occurred in August 2023. Results from pathogen testing from the second trip were not available at the time of writing, but lab work has begun. Phylogenetic analysis of the samples already tested found that none appeared to be betacoronaviruses, the lineage associated with previous highly pathogenic CoVs in human outbreaks (SARS-CoV, MERS-CoV, SARS-CoV-2). All were alphacoronaviruses and closely related to viruses previously found in bats in southeast Asia. It is likely that they represent endemic viruses circulating in these bat populations. None are known to be zoonotic or present a direct threat to human health, though generally they are uncharacterized and known only from short sequence fragments from similar testing in the past, but some of our samples matched most closely to viruses for which a whole genome sequence is available.

In addition to likely bat-associated CoVs, some food and surface samples tested positive for Infectious Bronchitis Virus (IBV), which is a common gammacoronavirus of poultry (see report 1.2.6.2 - Food water and surface sampling). Given that poultry (mostly chickens, though ducks in some cases) were common on the guano farms it is most likely that this represents evidence of poultry encountering the bat guano. Although the virus itself is not zoonotic, this finding is interesting as it tells us that livestock-associated RNA viruses are routinely carried into homes - likely either on the clothing or skin of people, though livestock entering the home is also possible in many cases. This means that livestock (and perhaps pets) could act as a mechanical vector, transporting contamination originating from bats or potentially act as bridging or mixing hosts -

by being infected with a bat-origin virus and transmitting it onward to humans. All three major CoV outbreaks (SARS, MERS, COVID-19) are either known or suspected to have involved an intermediate host rather than direct bat-human spillover transmission. Based on experience and given the highly divergent nature of bird vs. mammalian coronaviruses, a mammal seems more likely to be a high-risk intermediate host than a bird. Though the participating farmers have indicated informally that excluding domestic animals from the guano harvest area would be difficult, the STOP Spillover Cambodia social behavior change (SBC) team is continuing to explore the possibilities for risk reduction in this area.

While it is encouraging that neither previous published work, nor the present study have found known human pathogens nor any betacoronaviruses, the findings are nonetheless important. Alphacoronaviruses so far have only been associated with mild respiratory disease (cold-like symptoms) in humans, but some alphacoronaviruses do have the ability to infect humans and therefore the possibility of a harmful CoV outbreak associated with zoonotic alphacoronavirus transmission cannot be entirely ruled out. It will therefore be interesting to compare the present findings to the forthcoming surveillance testing findings from syndromic surveillance activities commencing shortly in the same area (Activity 2.2.2.2) to see whether syndromic targeted surveillance detects any human cases of infection with the same or similar viruses in people living in these communities.

Field and lab work on this activity will continue in project Year 4. The end of fieldwork is anticipated in February or March 2024 with lab work also scheduled for completion by the end of project Year 4. In-depth phylogenetic analysis as well as examination of seasonal patterns of shedding will only be possible once data collection is complete.

LITERATURE CITED

Amman, B. R. et al. "Seasonal pulses of marburg virus circulation in Juvenile *Rousettus aegyptiacus* bats coincide with periods of increased risk of human infection." *PLoS Pathog*. 8, 25 (2012).

Andersen, K. G., Rambaut, A., Lipkin, W. I., Holmes, E. C. & Garry, R. hF. "The proximal origin of SARS-CoV-2." *Nat. Med*. 26, 450–452 (2020).

Cappelle, J., Furey, N., Hoem, T., Ou, T. P., Lim, T., Hul, V., Heng, O., Chevalier, V., Dussart, P., & Duong, V. (2021). "Longitudinal monitoring in Cambodia suggests higher circulation of alpha and betacoronaviruses in juvenile and immature bats of three species." *Scientific Reports*, 11(1), Article 1. https://doi.org/10.1038/s41598-021-03169-z

Cappelle, J., Hoem, T., Hul, V., Furey, N., Nguon, K., Prigent, S., Dupon, L., Ken, S., Neung, C., Hok, V., Pring, L., Lim, T., Bumrungsri, S., Duboz, R., Buchy, P., Ly, S., Duong, V., Tarantola, A., Binot, A., & Dussart, P. (2020). "Nipah virus circulation at human-bat interfaces, Cambodia." *Bulletin of the World Health Organization*, 98(8), 539–547. https://doi.org/10.2471/BLT.20.254227

Corman VM, Landt O, Kaiser M, Molenkamp R, Meijer A, Chu DK, et al. "Detection of 2019 novel coronavirus (2019-nCoV) by real-time RT-PCR." *Euro Surveill*. 2020 Jan;25(3):2000045.

COVID-19 Weekly Epidemiological Update, 2023. WHO Coronavirus (COVID-19) Dashboard | WHO Coronavirus (COVID-19) Dashboard With Vaccination Data

Chhay, S. "Cambodian bats: a review of farming practices and economic value of lesser Asiatic yellow house bat Scotophilus kuhlii (Leach, 1821), in Kandal and Takeo provinces, Cambodia." *Cambodian Journal of Natural History*, 2012, 164–165.

Csorba, G., Furey, N. & Görföl, T. (2020) *Murina walstoni*. *The IUCN Red List of Threatened Species* 2020: e.T84562267A84562270. https://dx.doi.org/10.2305/IUCN.UK.2020-3.RLTS.T84562267A84562270.en. Accessed on 21 September 2023.

Drexler, J. F. et al. "Amplification of emerging viruses in a bat colony." *Emerg. Infect. Dis*. 17, 449–456 (2011). 10 Vol:.(1234567890) Scientific Reports | (2021) 11:24145 | https://doi.org/10.1038/s41598-021-03169-z www.nature.com/scientificreports/

Delaune, D., Hul, V., Karlsson, E. A., Hassanin, A., Ou, T. P., Baidaliuk, A., Gámbaro, F., Prot, M., Tu, V. T., Chea, S., Keatts, L., Mazet, J., Johnson, C. K., Buchy, P., Dussart, P., Goldstein, T., Simon-Lorière, E., & Duong, V. (2021). "A novel SARS-CoV-2

related coronavirus in bats from Cambodia." *Nature Communications*, 12(1), Article 1. https://doi.org/10.1038/s41467-021-26809-

Epstein, J. H. et al. "Pteropus vampyrus, a hunted migratory species with a multinational home-range and a need for regional management." *J. Appl. Ecol.* 46, 991–1002 (2009).

Mortlock, Marinda, Marike Geldenhuys, Muriel Dietrich, Jonathan H. Epstein, Jacqueline Weyer, Janusz T. Pawęska, and Wanda Markotter. "Seasonal shedding patterns of diverse henipavirus-related paramyxoviruses in Egyptian rousette bats." *Science report, Nature* 11:24262. 2021. https://www.nature.com/articles/s41598-021-03641-w.pdf.

Peel, A. J. et al. "The effect of seasonal birth pulses on pathogen persistence in wild mammal populations." *Proc. R. Soc. Lond. B Biol. Sci.* 281, 20132962 (2014).

Quan PL, Firth C, Street C, Henriquez JA, Petrosov A, Tashmukhamedova A, et al. "Identification of a severe acute respiratory syndrome coronavirus-like virus in a leaf-nosed bat in Nigeria." *mBio*. 2010 Oct 12;1(4):e00208-10.

Reynes, J.-M., Molia, S., Audry, L., Hout, S., Ngin, S., Walston, J., & Bourhy, H. "Serologic Evidence of Lyssavirus Infection in Bats, Cambodia." *Emerging Infectious Diseases*, 10(12), 2231–2234. 2004. https://doi.org/10.3201/eid1012.040459

USAID-PREDICT Cambodia, 2020: One Health In Action (2009-2020). https://static1.squarespace.com/static/5c7d60a711f7845f734d4a73/t/5f24d1a 3df814e31ef49c31f/1596248503271/FINAL+REPORT+COUNTRY-CAMBODI A-full.pdf

Wang, C., Horby, P. W., Hayden, F. G. & Gao, G. F. "A novel coronavirus outbreak of global health concern." *Lancet* 395, 470–473 (2020).

Wong, A. C. P., Li, X., Lau, S. K. P. & Woo, P. C. Y. "Global epidemiology of bat coronaviruses." *Viruses* 11, 174 (2019).

Woo, P. C. Y. et al. "Discovery of seven novel mammalian and avian coronaviruses in the genus deltacoronavirus supports bat coronaviruses as the gene source of alphacoronavirus and betacoronavirus and avian coronaviruses as the gene source of gammacoronavirus and deltacoronavirus." *J. Virol.* 86, 3995–4008 (2012).

Zhou, P. et al. "A pneumonia outbreak associated with a new coronavirus of probable bat origin." *Nature* 579, 270–273 (2020).

APPENDICES

APPENDIX 1: FIELD PROGRAM

Date	Activity	Participant
04-24-2023	a. Check the equipment and material for data collection<br>b. Travel to Khachav commune, Kang meas district, Kampong Cham<br>c. In the evening, cover plastic tarp for every 10 m^2 of used roosting area	OH-DReaM Working Group
04-25-2023	**Morning**<br><br>1. Early morning at 4 a.m. collect urine.<br><br>Place a 1m^2 plastic tarp for every 10 m^2 of used roosting area<br>○ This must be done while bats are roosting (during the day). Avoid perturbing the colony<br>○ Wait 20 minutes and collect all fresh samples found on the tarp. Pulling them (per tarp) on a 5ml conical tube using a swab or plastic disposable spoon<br>○ Add RNA shield in a ratio of 30:300 270 (for 30mg of feces add 300ìL of RNA shield solution)<br>○ Mix well, label and put on ice<br><br>2. Conduct interviews of 3-4 bat farm owners.<br>3. Take GPS location of farm (preferably at center of roosts)<br>○ Use waypoint averaging for high-precision location.<br>4. Take photos of the roost from each side<br>○ Focus on density of leaf bunches<br>5. Measure area cover of roosts<br>○ Estimate the % of area used by bats at the time of visit based on observation and discussion with the proprietor	OH-DReaM Working Group

6. Search roof area for bat carcasses, if found, take the photo, record the data with farm information and preserve in >90% ethanol

Evening (1 hour before sunset)

7. Installing Camera and infrared light.
    - Equipment needed
        i. Camera recorder with night vision mode (or security camera)
        ii. Infrared lights
        iii. Power source
    - Find a spot that will permit coverage of the greatest area of the roost
        i. If possible, set at least two cameras to cover different areas
        ii. Make sure wind is no more than a gentle breeze (leaves and twigs in motion), and it is not raining
    - Set camera and infrared light pointing to the roosts
        i. Position cameras to allow to have the bat silhouetted against the sky for easier viewing/recording

8. Set equipment for acoustic sampling 1 hour before sunset
    - Equipment needed
        i. SM4 FS detector with weatherproof microphone and cable lock
    - To sample bats emerging from roost, place detector 20m from (outside of) roost area (with a clear view of roost area) and direct microphone towards roost

04-26-2023	**Morning**<br><br>1. Collect urine sample<br>2. Continue interview bat farm owner (3-4 bat farmers)<br>3. Take GPS position<br>4. Take photo of roof from each side<br>5. Measure area cover of roots<br><br>**Evening (1 hour before sunset)**<br><br>1. Install camera and infrared light<br>2. Set equipment for acoustic sampling	OH-DReaM Working Group
04-27-2023	**Morning**<br><br>1. Collect urine sample<br>2. Continue interview bat farm owner (3-4 bat farmers) and three stakeholders from provincial, district and commune level<br>3. Take GPS position<br>4. Take photo of roof from each side<br>5. Measure area cover of roosts<br><br>**Evening (1 hour before sunset)**<br><br>1. Install camera and infrared light<br>2. Set equipment for acoustic sampling	OH-DReaM Working Group
04-28-2023	Travel back to Phnom Penh	

APPENDIX 2: QUESTIONNAIRE FOR OWNERS OF BAT GUANO FARMERS

I. General information

Code	Question	Answer	Skip
Q1	Interview code	__ __ __ __-__ __	
Q2	Location of interview	Province: ________________<br>District: ________________<br>Commune: ______________<br>Village: _________________	
Q3	Date of interview	DD__ __ MM __ __ YY__ __	
Q4	Interviewer		
Q5	Who is being interviewed?	• *(1)* Private<br>• *(2)* Community representative	2 -> Q8
Q6	What is owner's husband's name?		
Q7	What is owner's wife's name?		
Q8	What is a community representative's name?		
Q9	What is his/her phone number?		

Q10	Geolocation farms/harvest sites	Lon: ____________________<br>Lat: ____________________<br>Alt: ____________________	

II. Scale of bat guano farms/harvested sites

Code	Question	Answer	Skip
Roost occupancy (Proportional at a time point and variation over time)			
Q11	When did you start harvesting bat guano? (Year)		
Q12	Since starting to harvest bat guano, has there been a change in the number of bunches of roosts?	• *(0)* No<br>• *(1)* Yes	0 -> Q20
Q13	Did the number of bunches of roosts increase or decrease?	• *(0)* increase<br>• *(1)* decrease	
Q14	When did you start constructing bat roosts with concrete/wooden poles?		
Q15	How many bat roosts did you have in 2021?		
Q16	How many bat roosts did you have in 2020?		

Q17	How many bat roosts did you have in 2019?		
Q18	How many bat roosts did you have in 2018?		
Q19	How many bat roosts did you have in 2017?		
Q20	Do you have a plan to increase or decrease bat roosts on your farm next year?	• (0) No plan<br>• (1) Yes, I will increase<br>• (2) Yes, I will decrease	0 -> Q23
Q21	When will you increase or decrease them?		
Q22	Why do you have this plan?		
Quantity of bat guano harvested			
Q23	Are there changes in the quantity of bat guano harvested throughout a year?	• (0) No<br>• (1) Yes	0 -> Q26
Q24	When did you harvest the lowest quantity?		
Q25	When did you harvest the highest quantity?		

Q26	How many kilograms of bat guano can you harvest per week in the lowest period?		
Q27	How many kilograms of bat guano can you harvest per week in the highest period? (Or can you use from Jan. to March, April to June. I am concerned that farmers cannot remember!)		
Q28	In the last year, how many kilograms of bat guano have you harvested per week in the lowest period?		
Q29	In the last year, how many kilograms of bat guano have you harvested per week in the highest period?		
Bat species, density and fluctuations			
Q30	Do you know what bat species are living on your farms?	• (0) No<br>• (1) Yes	(0) -> Q32
Q31	What bat species are they?		
Q32	Approximately, how many bats are there on your farm?		
Q33	Are there fluctuations in the number of bats on your bat guano farms?	• (0) No<br>• (1) Yes	(0) -> Q38

Q34	When are there the lowest number of bats?		
Q35	Why are there the lowest number of bats?		
Q36	When are there the highest number of bats?		
Q37	Why are there the lowest number of bats?		
Q38	Is there a time of year when more dead bats are seen?	• (0) No<br>• (1) Yes	(0) ->Q41
Q39	If yes - why do you think this happens?		
Q40	Have you noticed an unusually large number of dead bats in a short period?	• (0) No<br>• (1) Yes	
Q41	If yes - why do you think that happened?		
Q42	Have you noticed other dead animals in the same period?	• (0) No<br>• (1) Yes	(0)->Q45
Q43	If yes - what species?		
Q44	If yes - why do you think?		

Q45	Do you see young bats in your roosts?	• (0) No<br>• (1) Yes	(0) ->Q47
Q46	When do you see them?		
Number of roosts and roost type			
Q47	How many bat roosts are there on the farms?		
Q48	How many bat roosts are constructed on trees? Ex: palm tree, coconut tree, etc.		
Q49	How many bat roosts are constructed on concrete/wooden poles?		
Q50	What type of each bat roost?	*Roost 1*<br>• (1) Constructed on tree<br>• (2) Constructed on concrete/wooden poles<br>*Roost 2*<br>• (1) Constructed on tree<br>• (2) Constructed on concrete/wooden poles<br>*Roost 3*<br>• (1) Constructed on tree<br>• (2) Constructed on concrete/wooden poles	

APPENDIX 3: CORRELATION ANALYSIS OF BAT GUANO PRODUCTION AND FARM SIZE

Variable	Mean	Bat guano production in the lowest period		
		P-Value	R^2	95% Interval Confidence
Roost area (m^2)	123.59	0.035	0.2799	0.005-0.130
Roost height (m)	4.81	0.697	0.01	–17.497 – 0.057
Number of bat roosts	13.18	0.003	0.47	0.312-1.235
