# Supplementary material for: Community Participatory Approach to Design, Test, and Implement Interventions That Reduce Risk of Bat-Borne Disease Spillover: A Case Study from Cambodia
Source: Trop Med Infect Dis. 2025 Dec 27;11(1):7. doi: 10.3390/tropicalmed11010007 (PMC12846364; doi:10.3390/tropicalmed11010007)
Supplement: Supplementary file 1 [file tropicalmed-11-00007-s001.zip › File S2. Cambodia Activity 1262_Final report of food water and surface contamination assessment.pdf]

Activity 1.2.6.2: Food, Water, and Surface Contamination Assessment in a Bat Guano Producing Community in Cambodia

A Report from STOP Spillover

September 2023

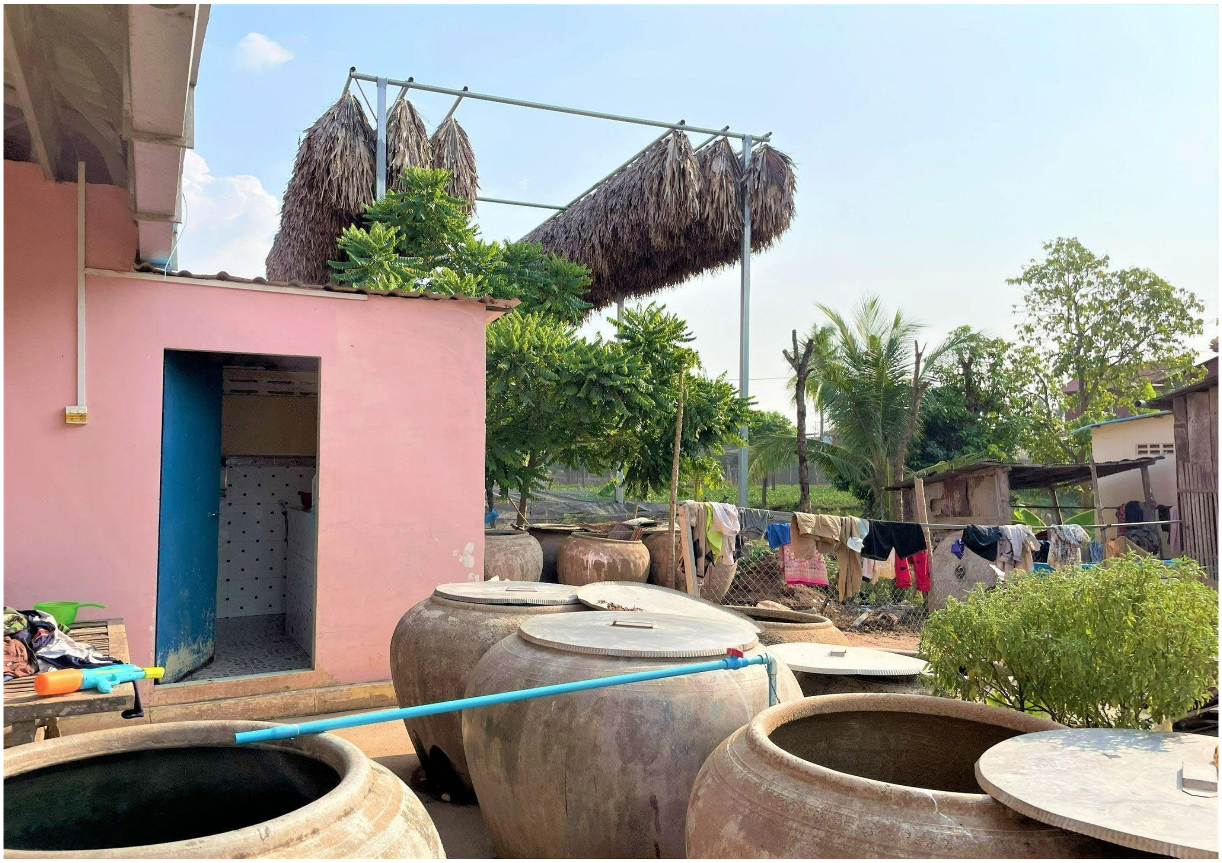

Photo Caption: A bat guano roost located behind a non-bat guano producing household and near a laundry/clothesline, a restroom, and open water containers in the high-risk interface. (Photo Credit: STOP Spillover Cambodia)

CONTENTS

List of Tables....i
List of Figures....i
List of Acronyms....ii
Executive Summary....iii
Introduction....1
Background....1
Objectives....3
Hypothesis....3
Methodology....4
Site selection....4
Sample size....5
Ethical approval....6
Household survey procedure....6
Sampling food, water, and household surfaces..6
Laboratory testing....9
Data analysis....10
Results....11
Discussion....20
Conclusions....23
Literature cited....24
Appendices....27
Appendix A: Survey Questionnaire....27
Appendix B: Survey Consent information sheet....48
Appendix C: Participant List for field survey/ sampling....49
Appendix D: Field program of SURVEY AND SAMPLING....50
Appendix E: LIST OF SURVEYED HOUSEHOLDS WITH SITE ID....52

STOP SPILLOVER

Strategies to Prevent Spillover (or "STOP Spillover") enhances global understanding of the complex causes of the spread of a select group of known zoonotic viruses from animals to humans. Funded by the United States Agency for International Development (USAID), the five-year project (2020 – 2025) builds government and stakeholder capacity to identify, assess, and monitor risks associated with known viruses. Target viruses include Ebola, Lassa, Marburg, Nipah, animal origin coronaviruses (including SARS, MERS-CoV, SARS-CoV-2), and animal origin influenza viruses such as highly pathogenic avian influenza. STOP Spillover will develop, test, and validate proven evidence-based risk reduction measures. In the context of this work "Spillover" refers to an event in which a zoonotic virus is transferred from a non-human animal host species (livestock or wildlife) to another animal or human. STOP Spillover is implemented in seven priority Asian and African countries.

This report is made possible by the generous support of the American people through USAID. The contents are the responsibility of STOP Spillover and do not necessarily reflect the views of USAID or the United States Government.

LIST OF TABLES

Table 1. Samples collected from Guano and non-guano-producing households 7

Table 2. Number of samples collected in each household 11

Table 3. Demographic and social characteristics of BGPHs and NBGPHs 12

Table 4. Sources, usage, storage of water in BGPHs and NBGPHs 13

Table 5. Food practices in BGPHs and NBGPHs 14

Table 6. Hygiene and sanitation practices in BGPHs and NBGPHs 15

Table 7. Bat exposure in BGPHs and NBGPHs 17

Table 8. Location and number of food, water, and surface samples detected positive for *Alphacoronavirus* and Infectious Bronchitis Virus in each of 20 bat and non-bat guano-producing households. 18

Table 9. Types of viruses detected in positive surface and food samples 19

LIST OF FIGURES

Figure 1. Map of Cambodia showing the geographic location of surveyed bat guano-producing community in Kang Meas district, Kampong Cham province. 5

Figure 2. Three different directions (horizontal, vertical and diagonal) of swabbing with the swab. 8

Figure 3. Sampling A water container, kitchen table and mango at bat and non-bat guano-producing households. (Photo Credit: USAID STOP Spillover) 8

Figure 4: Sampling drinking water and water used for vegetable gardening at bat and non-bat guano-producing households. (Photo Credit: USAID STOP Spillover) 9

LIST OF ACRONYMS

BGPH	Bat Guano Producing Household
GDAHP	General Directorate of Animal Health and Production
GHSAS	Global Health Security Agenda
IBV	Infectious Bronchitis Virus
IPC	Institut Pasteur du Cambodge
IRB	Tufts Health Sciences Institutional Review Board
JEE	Joint External Evaluation
NECHR	National Ethics Committee for Health Research of Cambodia
NGPH	Non-bat Guano Producing Household
OH DReaM WG	One Health Design, Research, and Mentorship Working Group
PDAFF	Provincial Department of Agriculture Forestry and Fisheries
PDE	Provincial Department of Environment
PHD	Provincial Health Department
PPE	Personal Protective Equipment
SBC	Social Behavior Change
SOP	Standard Operating Procedures
TIPs	Trials of Improved Practices

EXECUTIVE SUMMARY

Bat guano-producing communities face the very real possibility of viral contamination of food, water, and surfaces from viruses carried by bats. Discussions with key stakeholders in Kampong Cham province in July 2022 indicated that there is a knowledge gap regarding the role of food, water, and surface contamination coronavirus spillover risks. Participants were not aware that viruses could be transmitted from contaminated food, water, and surfaces at the bat-human interface. During plenary discussions with the bat guano-producer community, high-risk spillover activities were identified, including consuming water contaminated by bat guano or urine. Based on these discussions and previous desk research, the STOP Spillover team determined that an assessment of food, water, and surfaces in bat guano-producing communities was necessary to fill knowledge gaps related to understanding possible spillover routes.

The goal of this assessment was to understand community practices and behaviors that may increase the risk of human contact with bat-contaminated food and water. Data and findings from this assessment informed the design of interventions to reduce the risk of zoonotic virus spillover among communities living on or near bat guano farms. This assessment included the following activities: (i) identifying sampling sites, types of samples and the sampling protocol to use, through a desk review and consultative meetings with One-Health Design, Research and Mentorship (OH-DReaM) Working Group members and consortium experts; (ii) collecting samples from food, water, and other surfaces at bat guano-producing households and neighboring non-bat guano-producing households; (iii) testing and analyzing samples collected to examine for the presence of coronavirus RNA to quantitatively assess viral spillover risk from bats; and (iv) using assessment results to inform intervention design for community-level risk reduction interventions.

From April 24-27, 2023, the STOP Spillover Cambodia team, together with four OH-DReaM WG members and two consortium technical advisors, conducted a household survey paired with sampling of food, water, and household surfaces in bat guano-producing households in Kang Meas district, Kampong Cham province, to understand viral spillover risks and exposure pathways in bat guano-producing households and neighboring households. Over four days of sampling and survey data collection, 10 bat guano-producing households and 10 non-bat guano producing neighboring households were visited and interviewed, and 70 food samples, 75 water samples, and 376 household surface samples were collected for analysis.

Survey results and field observations determined that both bat and non-bat guano-producing households are close to bat roosts (less than 20 meters). Survey findings also showed a lack of water treatment and insufficient management or protection of water resources. Ninety percent of households did not cover drinking water storage containers, 55% did not cover containers of

water used for vegetable production, and 60% did not treat drinking water in their homes. In addition, 80% of surveyed households did not cover food and meat while drying it. Survey data and field observations suggested that improved hygiene and innovative food, water, and surface contamination interventions in bat guano-producing households are required to mitigate the risk of viral spillover from bats to humans.

Laboratory testing indicated that 1.4% of all food samples and 2.9% of all household surface samples were contaminated with *Alphacoronaviruses* and Infectious Bronchitis Virus (IBV). No water samples tested positive. Surface samples tested positive for potential bat-associated coronavirus RNA, whereas IBV RNA was found both outside and on interior surfaces, as well as on food surfaces. These findings suggest that priority community-level risk reduction interventions should focus on the disinfection of high-touch household surfaces, frequent hand washing, and covering foods left to dry or food stored in open containers in open kitchens.

Results from this study informed subsequent activities and interventions. The study identified risk factors for and levels of contamination on food and household surfaces. These findings will be used to monitor and validate the impact of trials of improved practices (TIPs), and social behavior change (SBC) interventions designed to support the adoption of improved practices. Combining findings from this study with household practices and behavior data will facilitate designing and implementing highly targeted interventions to reduce spillover risks through training, education, and awareness campaigns in bat-human interface communities and households.

INTRODUCTION

1.1 BACKGROUND

Strategies to Prevent (STOP) Spillover is a global initiative funded by USAID and is implemented in six countries in Africa and Asia, including Cambodia. The goal of STOP Spillover is to identify and reduce the risk of pandemic zoonotic virus spillover from animals (wildlife and domestic) into human populations. Human interactions with animals, and with the environment, are major drivers of spillover events. Bats host a diversity of viruses, including zoonotic viruses.

STOP Spillover's work includes engaging stakeholders in countries in Africa and Asia to explore the potential for zoonotic pathogenic viruses to transfer (or *spillover*) from animal reservoirs into humans. "Spillover" is a term referring to the transmission of a virus (or other pathogenic organism) from one species into a new one, as is believed to have occurred with Severe Acute Respiratory Syndrome Coronavirus 2 (SARS-CoV-2), the agent of coronavirus disease 2019 (COVID-19). Zoonotic spillover is a global threat. In many countries, the physical sites where spillovers occur are not known or completely understood. In addition, the input and knowledge of people and organizations closest to the interface where spillover occurs have often been overlooked.

The viral transmission pathways that cause pandemics include suspected spillover from animals, especially bats, due to the presence of many viruses in bats (Wang et al., 2018; Cui et al., 2019; Delaune et al., 2021). Increasingly, zoonotic (or animal-borne) viruses are considered to be important causative agents of waterborne, foodborne, and fomite (surface) infections throughout the world, making them a major concern for public health (Miranda & Schaffner, 2019; Ceylan et al., 2020; Yekta et al., 2021).

Viruses contaminating food, water, and surfaces lead to ongoing transmission of diseases. Viruses can contaminate food via three pathways: 1) contaminated water used to grow or process food, 2) direct contamination aided by poor hand hygiene practices, and 3) through the consumption of animal-based products containing zoonotic viruses (Le Guyader et al., 2008; Velebit et al., 2019). A number of serious food associated spillovers are either documented or suspected, most notably of Nipah and Hendra viruses via the consumption (by pigs and horses respectively) of fruits partially eaten by fruit bats (Field et al., 2001; Chua, 2003). In the case of Nipah virus, spillover also occurs directly from bats to humans via the consumption of contaminated raw palm sap (Islam et al., 2016). Community drinking water sources are also at risk of contamination from human and animal waste, as indicated by studies showing that feces, urine, and sputum carry SARS-CoV-2 (Purnama & Susanna, 2020; Bilal et al., 2020).

Besides food and water, surfaces are virus carriers. Different types of surfaces are reported to be contaminated with SARS-CoV-2 in healthcare and household settings (Döhla et al., 2020; Wong et al., 2020), workplaces (Marshall et al., 2020), laboratories (Bloise et al., 2020; Lv et al.,2020) and outdoors (Lee et al., 2020; Hu et al., 2020; Jiang et al., 2020). Household surfaces contaminated with SARS-CoV-2 included hard furniture, electronics, beds, floors, fabric, bathroom, and food/utensils (Döhla et al., 2020; Wong et al., 2020). Research demonstrates that SARS-CoV-2 can survive on surfaces such as nitrile gloves for a half-life of 2.26-15.33 hours, ABS plastic for a half-life of 2.26-15.33 hours, and stainless steel for a half-life of 2.26-17.9 hours (Fomenko et al., 2020; Biryukov et al., 2020; Kratzel et al., 2020; van Doremalen et al., 2020; Liu et al., 2020). These data suggest that SARS-CoV-2 can be fairly stable in ambient environmental conditions. While surface contamination is not believed to be the primary transmission route for SARS-CoV-2, other viruses are routinely transmitted by surface contamination (Sattar et al 1986).

In Cambodia, contact between bats and humans has been identified as a spillover risk. Opportunities for food-borne, water-borne, and fomite transmission of viruses—including coronaviruses—are high in bat guano producer communities. Work by USAID PREDICT Cambodia revealed that people living in Kang Meas district, Kampong Cham province (a community where the construction of artificial roosts for bat guano harvest is common), consumed uncovered food and water from unprotected sources that could be contaminated with bat droppings and urine (USAID, 2020). In Kang Meas district, some households obtain their drinking water from uncovered sources including rainwater cisterns, wells or ponds, which could contain infectious materials contaminated by bats (USAID, 2020). In addition, results from community level consultations with STOP Spillover staff demonstrated a knowledge gap regarding the possibility of food-borne, water-borne, and fomite viral infection. Stakeholders were not aware of this possible contamination and transmission pathway in and around their homes. Therefore, an assessment of food, water, and surface contamination risks in bat guano-producing communities is necessary to fill knowledge gaps related to understanding possible transmission routes via food, water, and surfaces. In this study, we tested common household surfaces and food surfaces, as well as household water sources for the presence of bat guano, and specific viral indicators of spillover risk.

This activity contributes to Global Health Security Agenda (GHSA) priorities focused on the surveillance of zoonotic diseases (P5.1) and risk communication (R5.1) and strengthens Cambodia's Joint External Evaluation (JEE) scores related to zoonotic disease surveillance, coordination, and private sector engagement (for food and water safety).

1.2 OBJECTIVES

This assessment used information from laboratory tests and surveys to identify priority interventions to reduce spillover risk from bat guano along the food, water, and surface spillover pathways in bat guano producing communities. Assessment objectives were:

- Determine the presence of coronavirus RNA in food, water, and surfaces in bat guano producing communities, to quantitatively assess viral spillover risk from bats.
- Fill knowledge gaps related to food, water, and surface management practices related to spillover risk.

1.3 HYPOTHESIS

The hypothesis of this study was:

- Some food, water, and surfaces in bat guano-producing communities and households are contaminated with bat guano, and we can measure that contamination as an indicator of spillover risk.

METHODOLOGY

The food, water, and surface study was conducted at the bat-human interface in Khchau commune, Kang Meas district, Kampong Cham province. Fieldwork and data collection took four days. Household interviews and sampling were conducted to understand contamination levels and sources on food, water, and surfaces in bat guano-producing households and non-bat guano producing households in the same community. Four One Health Design, Research and Mentoring Working Group (OH-DReaM WG) members from national and subnational levels including the General Directorate of Animal Health and Production (GDAHP), the Provincial Department of Agriculture Forestry and Fisheries (PDAFF), the Provincial Health Department (PHD) and the Provincial Department of Environment (PDE), two STOP Spillover consortium experts from Tufts University, and two STOP Spillover Cambodia Country Team members participated in data collection. Everyone participating in this research was trained on Personal Protective Equipment (PPE) requirements, survey tools, and in the Standard Operating Procedures (SOP) for sampling and household survey practices by STOP Spillover technical advisors and Country Team members.

2.1 SITE SELECTION

In June 2022, a national stakeholder engagement meeting was held to discuss zoonotic spillover risks in Cambodia, and to prioritize the interface, pathogen, and geographical location for STOP Spillover interventions in Cambodia. The bat-human interface in Kampong Cham province was prioritized by national stakeholders for STOP Spillover research and implementation in Cambodia. The bat-human interface in Kampong Cham province includes bat guano-producing communities located in the central lowlands of the Mekong River in south-eastern Cambodia (Khchau commune, Kang Meas district; Figure 1). Overall, 17 bat guano producing households were identified for this assessment.

FIGURE 1. MAP OF CAMBODIA SHOWING THE GEOGRAPHIC LOCATION OF SURVEYED BAT GUANO-PRODUCING COMMUNITY IN KANG MEAS DISTRICT, KAMPONG CHAM PROVINCE.

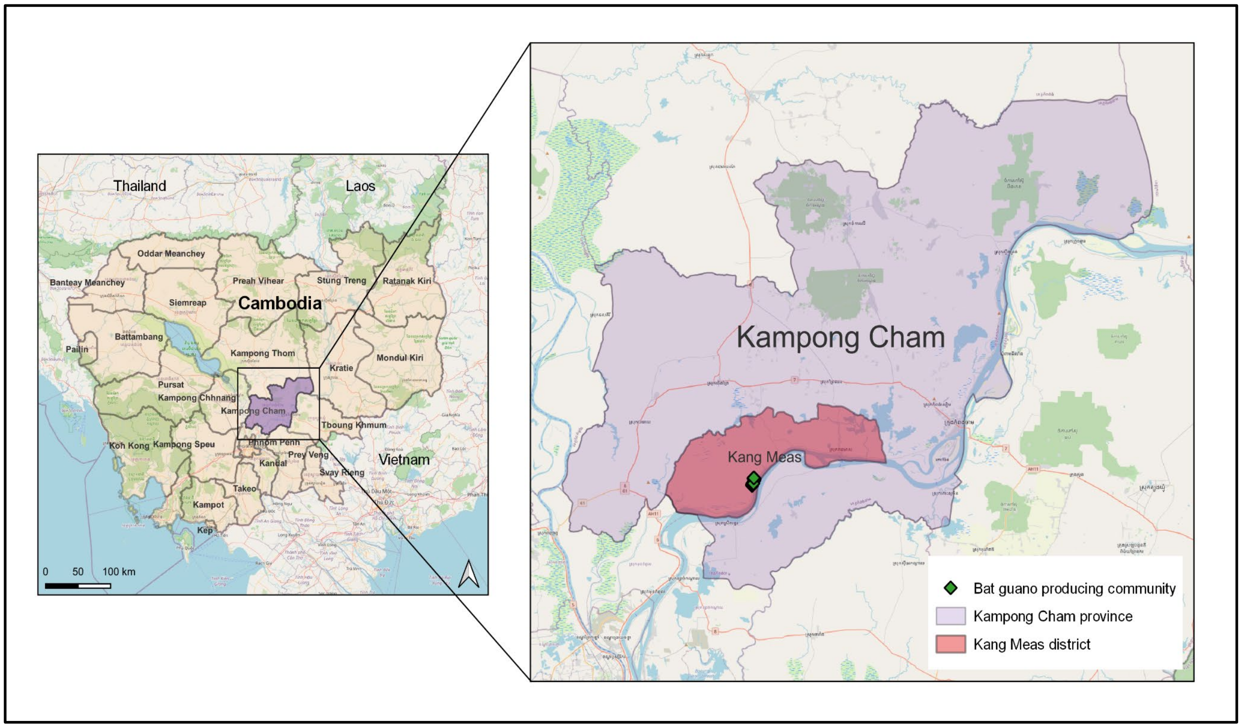

2.2 SAMPLE SIZE

The study population included 10 bat guano-producing households and 10 non-bat guano-producing households in a guano-producing region of Cambodia. The number of households sampled was determined based on laboratory capacity, funding available, time, and the number of households required to see variation across households in the community. Households were selected for general representation. Households headed by people of at least 18 years of age were approached to participate. With their informed consent: 1) swab samples from food and household surfaces and a water sample were collected from each household; and 2) a short survey conducted with the head of household (male or female) was conducted.

Ten bat guano-producing households were selected from the total number of all bat guano-producing households (17 households) using the following mechanisms:

- Observation of their activities in bat guano production adjacent to their houses.
- Input from local authorities.
- Their participation in a previous study in this project (approved by the Tufts I SBER IRB).

Ten non-bat guano-producing households were selected nearest to the bat guano-producing households that consented to be included in the study.

2.3 ETHICAL APPROVAL

The study protocol was approved by the Tufts Health Sciences Institutional Review Board (IRB; IRB ID is STUDY00003488). The protocol was also approved by the National Ethics Committee for Health Research (NECHR) of Cambodia. Enumerators received ethics and compliance training before data collection. All participants provided informed consent and were informed that participation was voluntary and that they could withdraw at any time.

2.4 HOUSEHOLD SURVEY PROCEDURE

Enumerators (OH-DReaM WG members) were trained, oriented, and supervised by STOP Spillover consortium technical advisors and STOP Spillover Country Team members. OH-DReaM WG members visited bat guano producing communities to conduct individual household interviews with respondents from bat guano-producing households and non-bat guano-producing households. Enumerators used a questionnaire (attached as Appendix A) related to food, water, and surface hygiene in their households. Household members were asked if they would be willing to participate. All heads of households (74% female, 26% male) were provided a verbal explanation of the purpose of the survey, which included a discussion about participants' right to privacy, and the right to withdraw from the interview at any time. No interview was conducted unless the individual head of household understood the purpose and process of the survey. The verbal informed consent form is described in Appendix B. The questionnaire took approximately 60 minutes for both guano- producing and non-guano-producing households. Global positioning system (GPS) data was collected from each household using waypoint averaging. Survey results were recorded on paper and entered into Microsoft Excel® for analysis.

2.5 SAMPLING FOOD, WATER, AND HOUSEHOLD SURFACES

Participating OH-DReaM WG members were trained and supervised by STOP Spillover consortium technical advisors and STOP Spillover Cambodia Country Team members for sample collection before field sampling took place. Food, water, and household surface samples were collected during the daytime, at the same time as the household survey. Participants were asked if they agreed to allow samples of food, water, and surfaces in their houses to be collected. The STOP Spillover team collected verbal informed consent for both the household survey and surface sampling. Masks and gloves were used as PPE before entering each bat-guano-producing household to collect food, water, and surface samples.

The sampling process was conducted inside and outside of participant homes. At each household, a map was hand-drawn of the household, and sample locations were selected based

on prioritized food, surface, and water sources including those with a high risk of spillover (Table 1).

TABLE 1. SAMPLES COLLECTED FROM GUANO AND NON-GUANO-PRODUCING HOUSEHOLDS

Type of sample	Prioritized samples collected
Food	Dried banana, coconut waste, dried fish, dried pork, green vegetables (leftovers), jackfruits, leftover fish, leftover pork, mango, orange, potato, raw meat, rice, sugar cane, tomato, and vegetable waste/garbage
Surface	Bat roosts, basket over food, ceramic container for bat guano, clothes (near bat roost, in and out house), cooking table, cover on rice, hat for collecting bat guano, outside table, plate (inside in kitchen uncovered and outside kitchen), railing, table, fridge, table near stove, toilet door, upstairs floor, upstairs table, and water containers (outside and in the kitchen)
Water	Drinking water and water used for vegetable gardening

A total of 521 food, water, and household surface samples were collected from bat and non-bat guano producing households (70 food samples, 75 water samples and 346 surface samples). All samples were duplicated.

Methods for each sampling type (food, surface, and water) are presented below. Supplies used for food and surface sample collection included sample swabs, cryovials, plastic stencils, coolers, permanent markers, hand sanitizer, gloves (while conducting sampling), masks (while inside houses), alcohol towelettes for stencil disinfection, bleach wipes for stencil disinfection, pens, paper towels, and observation guides / survey tools. Using the observation guide, a household plan was drawn, and the number and location of surfaces to be sampled was indicated. Depending on the type of samples selected, a permanent marker was used to label the cryovials, including the sample identification number, the collection time, and the type (source) of each sample.

Food and household surface samples

The procedure for sampling food and household surfaces with a swab can be described as follows:

- Disinfect hands with hand sanitizer.
- Disinfect the stencil with bleach and then an alcohol towelette.
- Place the stencil on the surface to be sampled.

- Open the tube containing the cotton swab carefully, making sure not to touch the tip of the swab to any surface or hands. Swab the surface of the sample item and put the swab into a cryovial tube prefilled with 0.9ml of DNA/RNA Shield™.
- Traverse the area inside the stencil in a systematic way, swabbing in three different directions (horizontal, vertical, diagonal), with the swab. Typically, it takes 10 swipes in each direction to cover the surface area of the stencil. Go slowly and steadily using a slight rolling motion so all sides of the tip are in contact with the surface (Figures 2 and 3).
- Replace the swab in the solution tube, close it carefully, tighten the lid, and place it in the cooler on ice to be returned to the laboratory.
- Label sample tubes using a code that includes country, activity, year, sample type, site ID and sample number.
- Disinfect the stencil with another moist towelette.

FIGURE 2. THREE DIFFERENT DIRECTIONS (HORIZONTAL, VERTICAL AND DIAGONAL) OF SWABBING WITH THE SWAB.

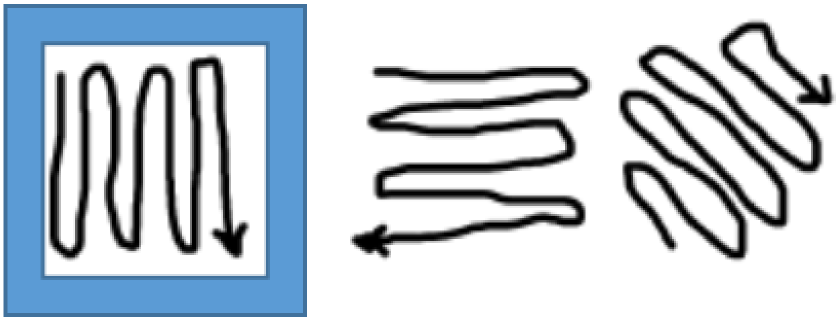

FIGURE 3. SAMPLING A WATER CONTAINER, KITCHEN TABLE AND MANGO AT BAT AND NON-BAT GUANO-PRODUCING HOUSEHOLDS. (PHOTO CREDIT: USAID STOP SPILLOVER)

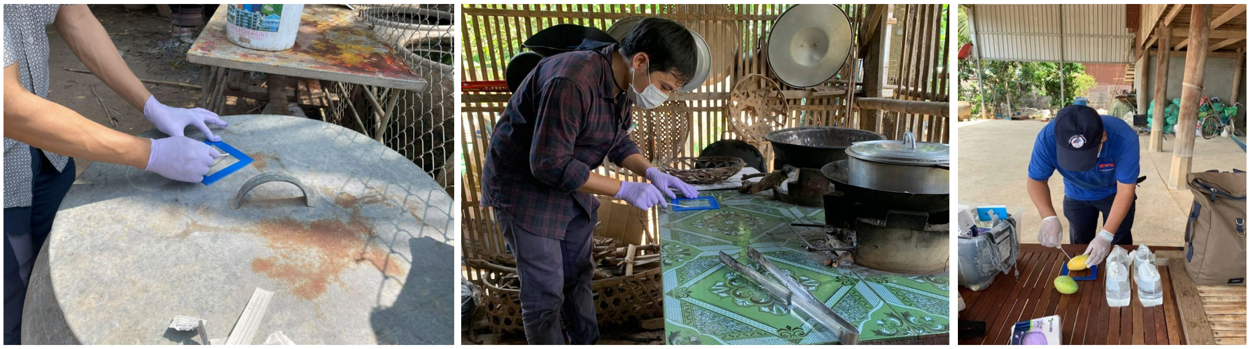

Water samples
Water samples were collected using a Whirl-Pak® bag (0.5L), garbage bags, absorbent pads, a cooler with ice, permanent markers, hand sanitizer, survey forms, gloves (while sampling), and masks (while inside houses). The procedure for water sampling is described as follows:

- Take a new Whirl-Pak® bag and label it with a permanent marker including date, country code, activity, year and sample type, site ID, and sample number.
- Disinfect hands.
- Rip the top plastic off at the perforation and place it in your bag. Open the bag by pulling the white tabs apart. Do not touch inside the bag or blow into the bag to get it to open.
- Pour the sample into the Whirl-Pak® bag without touching the lip of the bag. Volume to be collected: 0.5 to 1 L.
- Whirl the bag closed, twist the white metallic wire securely, and gently invert to check there are no leaks.
- Place the Whirl-Pak® bag upright in the cooler on ice for return to the laboratory.
- Pack the sample bags inside a garbage bag, then place this bag in a second bag. Between the two bags, place one absorbent sheet per 0.5L bag. Note: The absorbent sheets can be reused if they are not excessively wet.
- Twist the two bags tightly and secure them with tape or a cable tie once all samples are loaded, before transporting to the lab.
- Store at <10°C until samples reach the laboratory at IPC.
- Ship to diagnostic lab within 24 hours after collecting either by licensed freight carrier or hand-carry by project-affiliated personnel.

FIGURE 4: SAMPLING DRINKING WATER AND WATER USED FOR VEGETABLE GARDENING AT BAT AND NON-BAT GUANO-PRODUCING HOUSEHOLDS. (PHOTO CREDIT: USAID STOP SPILLOVER)

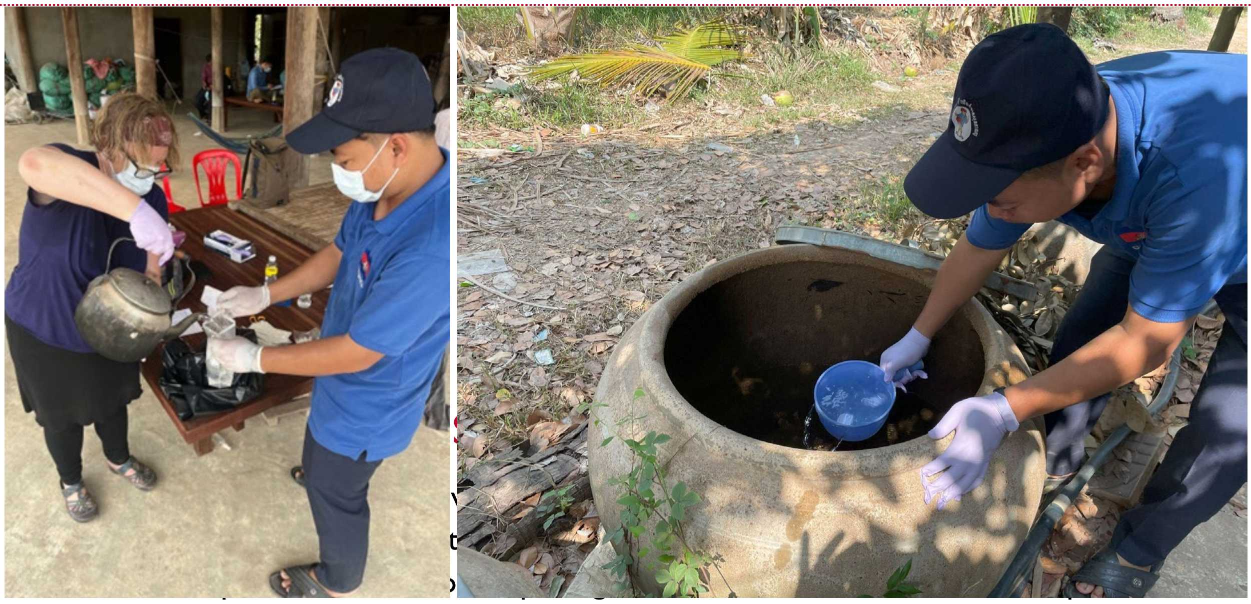

normal ice to the laboratory to detect the presence of coronavirus RNA. All samples collected were transferred to IPC for detection of coronavirus RNA using PCR/RT-PCR and sequencing. This testing measured general spillover risk, as opposed to the infectivity of a particular virus. With this general spillover risk measured, more effective evidence-based interventions to prevent spillover can be developed and implemented.

Coronavirus testing

First, water samples were filtered. For water filtration, 100 milliliters of water were transferred into a vacuum-driven filtration system using a Stericup® (Millipore Sigma, St Louis MO, USA). Water flowed through a 0.45µm PVDF membrane filter supported by a filter support. After that, the membrane filter was removed and placed on a plate containing the appropriate medium. RNA was extracted from surface swabs using the Zymo Research Direct-zol™ RNA MiniPrep kit (Zymo Research, CA, USA; https://zymoresearch.eu/products/direct-zol-rna-miniprep-kits). A SuperScript™ III First-Strand Synthesis SuperMix was used to synthesize cDNA (https://www.thermofisher.com/order/catalog/product/11904018)(Catalog 12574026; Invitrogen first strand cDNA).

To detect SARS-CoV-2 RNA, E & N gene assays were utilized. The protocol included the E-sarbeco-F1, E-sarbeco-R2 primers and E-Sarbeco-P1-FAM (*Corman et al., 2020, https://doi.org/10.2807/1560-7917.ES.2020.25.3.2000045*), as well as HKU-NF, HKU-RN primers and HKU-NP-Cy5 (*by Hong Kong U, https://www.who.int/docs/default-source/coronaviruse/whoinhouseassays.pdf*) following reverse transcription with Superscript III RT/Platinum.

To detect coronavirus RNA, conventional PCR was utilized. The protocol adapted from Quan PL et al. 2010 (10.1128/mBio.00208-10) used SuperScript™ III First-Strand Synthesis SuperMix, with catalog number is 18080-400 for cDNA synthesis and Invitrogen™ Platinum™ Taq DNA Polymerase, with the catalog number 10966-026 for amplification (https://www.thermofisher.com/order/catalog/product/15966005). PCR products were visualized by agarose gel electrophoresis. Suspect positive products were subject to Sanger sequencing by a commercial laboratory (Macrogen, Seoul, South Korea). Products were sequenced in both directions and consensus sequences reported.

2.6 DATA ANALYSIS

Survey data were collected on paper and entered into Microsoft Excel® for cleaning. Results of coronavirus testing were also entered into Microsoft Excel®. Initial phylogenetic sequence analysis was performed using GenBank® nucleotide blast search.

RESULTS

3.1 RESULTS

OH-DReaM WG members collected food, water, and surface samples, and conducted the household survey in bat guano producing communities. The household survey was conducted with 10 bat guano-producing households (BGPH) and 10 non-bat guano neighbor households (NBGPH). At the same time, 70 food samples, 75 water samples, and 376 surface samples were collected from the surveyed households. The detailed number of samples collected from each household is shown in Table 2.

TABLE 2. NUMBER OF SAMPLES COLLECTED IN EACH HOUSEHOLD

Household ID	Number of samples			Total samples
	Food	Surface	Water	
**10 BGPH**				
BGPH1	0	26	4	30
BGPH4	4	20	4	28
BGPH6	4	24	4	32
BGPH9	4	18	4	26
BGPH10	2	20	4	26
BGPH11	4	22	4	30
BGPH12	2	22	4	28
BGPH14	0	20	4	24
BGPH15	6	20	4	30
BGPH16	4	16	4	24
**10 NBGPH**				
NBGPH1	2	18	4	24
NBGPH4	2	14	2	18
NBGPH6	8	16	3	27
NBGPH9	8	20	4	32
NBGPH10	4	16	4	24
NBGPH11	4	16	4	24
NBGPH12	0	18	4	22
NBGPH14	4	18	2	24
NBGPH15	6	16	4	26
NBGPH16	2	16	4	22
Total	70	376	75	521

3.2 HOUSEHOLD SURVEY

6.76.1 General information from BGPHs and NBGPHs

The household survey was completed with 20 households (10 BGPHs and 10 NBGPHs) in Khchau commune, Kang Meas district, Kampong Cham province, and detailed demographic and social characteristics of BGPHs and NBGPHs are shown in Table 3. All respondents were heads of households; 74% were women. The average respondent age was 56.7 years. Most heads of households could read (80% female, 89% male), and most respondents (80%) had attended school. Wired electricity was present in all BGPHs and NBGPHs. House construction varied—all BGPHs and 50% of NBGPHs had wood walls, 80% of BGPHs and 60% of NBGPHs had dirt floors, and 80% of BGPHs and 70% of NBGPHs had tile roofs. Additionally, the average number of sleeping places reported by respondents was 3 for BGPHs and 3.3 for NBGPHs.

TABLE 3. DEMOGRAPHIC AND SOCIAL CHARACTERISTICS OF BGPHS AND NBGPHS

	BGPHs (N = 10)		NBGPHs (N = 10)		Overall (N = 20)	
N(%) Female respondents	6/9	(67%)	8/10	(80%)	14/19	(74%)
Mean (sd) respondent age in years	61.7	(7.04)	51.7	(22.0)	56.7	(16.7)
N(%) Respondent attended school	8/10	(80%)	8/10	(80%)	16/20	(80%)
Mean (sd) Respondent number of years of school completed	5.5	(2.5)	6.5	(4.2)	6	(3.38)
N(%) Female head of household can read	7/10	(70%)	9/10	(90%)	16/20	(80%)
N(%) Male head of household can read	9/10	(90%)	8/9	(89%)	17/19	(89%)
N(%) House with wood wall	10/10	(100%)	5/10	(50%)	15/20	(75%)
N(%) House with dirt floor	8/10	(80%)	6/10	(60%)	14/20	(70%)
N(%) House with tile roof	8/10	(80%)	7/10	(70%)	15/20	(75%)
N(%) Household with wired electricity	10/10	(100%)	10/10	(100%)	20/20	(100%)
Mean (sd) number of sleeping places	3	(1.05)	3.3	(0.82)	3.15	(0.93)

Abbreviation: Bat guano-producing household (BGPH), Non-bat guano-producing household (NBGPH), Standard Deviation (sd)

3.2.2 Water practices

Sources, usage, and storage of water in BGPHs and NBGPHs are presented in Table 4. Household taps were reported to be the most used source for drinking water at 80% of BGPHs and 50% of NBGPHs. Overall, 40% of respondents reported collecting non-drinking water from protected wells. Additionally, 20% of BGPHs and NBGPHs believe the current water in the house is safe to drink. Most respondents (100% BGPHs and 80% NBGPHs) reported covering water storage containers in their households. BGPHs (70%) were more likely to treat their water than NBGPHs (50%). Boiling water was used by all households that reported treating water in their home. All respondents from BGPHs and NBGPHs reported using water to grow vegetables; 70% of BGPHs and 40% of NBGPHs had covers for water storage containers used for vegetable production.

TABLE 4. SOURCES, USAGE, STORAGE OF WATER IN BGPHS AND NBGPHS

	BGPHs (N = 10)		NBGPHs (N = 10)		Overall (N =20)	
N(%) Reported drinking water sources where households collect most often						
Kiosk or tap not at the house	0	(0%)	1	(10%)	1	(5%)
Tap at the house	8	(80%)	5	(50%)	13	(65%)
Purchased bottle/bag	0	(0%)	4	(40%)	4	(20%)
Rainwater	1	(10%)	0	(0%)	1	(5%)
Mixed sources	1	(10%)	0	(0%)	1	(5%)
Mean (sd) time per day drinking water is collected (minutes)	2.7	(0.48)	2.81	(0.98)	2.75	(0.76)
N(%) Reported collecting non-drinking Water from protected well	5	(50%)	3	(30%)	8	(40%)
N(%) Respondent believes current water in the house is safe to drink	2	(20%)	2	(20%)	4	(40%)
N(%) Reported types of storage container for safe drinking water						
Metal pot	2	(20%)	1	(10%)	3	(15%)
Bucket	6	(60%)	7	(70%)	13	(65%)
Barrel	3	(32%)	3.2	(32%)	6.4	(32%)
N(%) Reported covering storage containers of water	10	(100%)	8	(80%)	18	(90%)
N(%) Reported treating water at sources	6	(60%)	4	(40%)	10	(50%)
N(%) Reported treating water in the house	7	(70%)	5	(50%)	12	(60%)
N(%) Reported treating the water in the house using boiling method	7/7	(100%)	5/5	(100%)	12/12	(100%)
N(%) Reported using water for vegetable gardening	10	(100%)	10	(100%)	20	(100%)
N(%) Reported covering water storage containers used for vegetable gardening	7	(70%)	4	(40%)	11	(55%)

Abbreviation: Bat guano-producing household (BGPH), Non-bat guano-producing household (NBGPH), Standard Deviation (sd).

3.2.3 Food practices

Survey results on food consumption in BGPHs and NBGPHs are presented in Table 5. Water was the most consumed beverage for all BGPHs and NBGPHs, followed by tea and coffee (100% BGPHs and 60% NBGPHs). All respondents from BGPHs and NBGPHs reported consuming cooked vegetables, meat and cereal, while most respondents (90% BGPHs and 100% NBGPHs) reported eating fruits, vegetables and cooked fish over the past week. All BGPHs and NBGPHs reported washing fruits and vegetables before eating. Overall, 95% of households used individual plates, and respondents from all BGPHS and NBGPHs use silverware/utensils when eating. For food storage, a refrigerator was used by 30% of BGPHs and 40% of NBGPHs for

keeping food, followed by bags (20% BGPHs and 40% NBGPHs) and cool boxes (30% BGPHs and 10% NBGPHs). Of the total respondents, 15% think they can get sick from food. At the time of the survey, 15% had no food stored in their home; 5% believed food in their houses was safe to eat, while 80% did not know if food in their houses was safe to eat. Most (80%) of BGPHs and NBGPHs reported drying food or meat outside their houses, and all respondents who reported drying food reported they did not cover their food or meat while drying it.

TABLE 5. FOOD PRACTICES IN BGPHS AND NBGPHS

	BGPHs (N=10)		NBGPHs (N=10)		Overall (N=20)	
N(%) Reported types of drinks during the day						
Soda	0	(0%)	3	(30%)	3	(15%)
Water	10	(100%)	10	(100%)	20	(100%)
Tea-Coffee	10	(100%)	6	(60%)	16	(80%)
Milk	0	(0%)	1	(10%)	1	(5%)
Juice	5	(50%)	5	(50%)	10	(50%)
N(%) Reported types of food eaten over the past week						
Fruit	9	(90%)	10	(100%)	19	(95%)
Vegetables	9	(90%)	10	(100%)	19	(95%)
Cooked vegetables	10	(100%)	10	(100%)	20	(100%)
Meat	10	(100%)	10	(100%)	20	(100%)
Fish cooked	9	(90%)	10	(100%)	19	(95%)
Cereal	10	(100%)	10	(100%)	20	(100%)
Dairy	7	(70%)	3	(30%)	10	(50%)
N(%) Reported washing fruits and vegetables before eating	10	(100%)	10	(100%)	20	(100%)
N(%) Household uses individual plates when eating	10	(100%)	9	(90%)	19	(95%)
N(%) Household use silverware/utensils	10	(100%)	10	(100%)	20	(100%)
N(%) Household purchased prepared food over the past week	2	(20%)	4	(40%)	6	(30%)
N(%) Reported types of storage that household used to keep food						
Fridge	3	(30%)	4	(40%)	7	(35%)
Bag	2	(20%)	4	(40%)	6	(30%)
Cool box	3	(30%)	1	(10%)	4	(20%)
N(%) Respondent thinks they can get sick from food	2	(20%)	1	(10%)	3	(15%)
N(%) Respondent believes food is safe to eat						

	BGPHs (N=10)		NBGPHs (N=10)		Overall (N=20)	
N(%) Respondent believes foods in the house are safe to eat	1	(10%)	0	(0%)	1	(5%)
N(%) Respondent do not know if foods in their houses are safe to eat	7	(70%)	9	(90%)	16	(80%)
N(%) Respondent has no food at the time of the survey	2	(20%)	1	(10%)	3	(15%)
N(%) Reported having seen animal feces in or near food in the house	1	(10%)	1	(10%)	2	(10%)
N(%) Reported drying meat outside the house	8	(80%)	8	(80%)	16	(80%)
N(%) Household member has not covered food and meat while drying	8	(80%)	8	(80%)	16	(80%)

Abbreviation: Bat guano-producing household (BGPH), Non-bat guano-producing household (NBGPH)

3.2.4 Hygiene and sanitation practices

Table 6 shows survey results related to sanitation and hygiene practices in BGPHs and NBGPHs. Overall, 90% of households reported they have dedicated cooking places inside their houses. Respondents from 90% of BGPHs and 70% of NBGPHs showed dedicated space for dishwashing inside their houses to the enumerator; soap (100% of respondents) and sponges (95% of respondents) were used for washing dishes. Half of all houses surveyed reportedly clean their floor daily, and 70% reported they use a wet mop with detergent to clean the floor.

Overall, 80% of BGPHs and 100% of NBGPHs reported using individual household latrines, and soap and brushes were reportedly used by all respondents for cleaning the latrine floor. For hand washing, only 2% of BGPHs had a dedicated space for washing hands, but all BGPHs and NBGPHs reported the use of soap to wash hands. Overall, 20% of respondents reported using running water, and 75% of respondents reported using water in containers to wash their hands. Regarding trash practices, bags were reported to be used for storing trash by most households (100% BGPHs and 90% NBGPHs), and all households reported burning their trash after collection.

TABLE 6. HYGIENE AND SANITATION PRACTICES IN BGPHS AND NBGPHS

	BGPHs (N=10)		NBGPHs (N=10)		Overall (N=20)	
N(%) Household has dedicated cooking place inside the house	9	(90%)	9	(90%)	18	(90%)
N(%) Household has dedicated space for dishwashing inside the house	9	(90%)	7	(70%)	16	(80%)
N(%) Reported washing dishes after every meal	7	(70%)	10	(100%)	17	(85%)
N(%) Reported types of materials used for washing dishes						

	BGPHs (N=10)		NBGPHs (N=10)		Overall (N=20)	
Soap	10	(100%)	10	(100%)	20	(100%)
Sponge	9	(90%)	10	(100%)	19	(95%)
N(%) Reported cleaning floor in the house daily	5	(50%)	5	(50%)	10	(50%)
N(%) Reported using wet mop with detergent for cleaning floor	7	(70%)	7	(70%)	14	(70%)
N(%) Reported doing laundry daily	9	(90%)	10	(100%)	19	(95%)
N(%) Household has dedicated space for laundry inside the house	3	(30%)	4	(40%)	7	(35%)
N(%) Reported using detergent and soap for cleaning clothes	10	(100%)	10	(100%)	20	(100%)
N(%) Household has shared latrine	8	(80%)	10	(100%)	18	(90%)
N(%) Reported cleaning floor of The latrine sometimes	9	(90%)	10	(100%)	19	(95%)
N(%) Reported using soap and brush for leaning floor of latrine	10	(100%)	10	(100%)	20	(100%)
N(%) Household has dedicated space for hand washing place	2	(20%)	0	(0%)	2	(10%)
N(%) Reported using soap present for washing hands	10	(100%)	10	(100%)	20	(100%)
N(%) Reported type of water used for washing hands						
Running water	3	(30%)	1	(10%)	4	(20%)
Water in container	7	(70%)	8	(80%)	15	(75%)
N(%) Reported using bag for storing trash	10	(100%)	9	(90%)	19	(95%)
N(%) Reported burning trash	10	(100%)	10	(100%)	20	(100%)
N(%) Reported daily trash collection	2	(20%)	2	(20%)	4	(20%)

Abbreviation: Bat guano-producing household (BGPH), Non-bat guano-producing household (NBGPH)

3.2.5 Bat exposures

All participants were also surveyed on disturbances from bats and bat roost condition in their households (Table 7). All BGPHs and 60% of NBGPHs are located less than 20 meters from the bat roosts. Among all households, only 10% of NBGPHs reported that bats are living in the house and experienced disturbances from bats, such as bats entering the house, water contamination by bats, the smell of bat urine and guano, bat excreta, bat noise and fear of disease. The average number of bat roosts in BGPHs surveyed is 3.5. Of all BGPHs, 80% constructed bat roosts on concrete/wooden poles, and 20% constructed bat roosts on trees.

TABLE 7. BAT EXPOSURE IN BGPHS AND NBGPHS

	BGPHs (N = 10)		NBGPHs (N = 10)		Overall (N = 20)	
N(%) Reported there are bats living in the house	0	(0%)	1	(10%)	1	(5%)
N(%) Reported distance 1-20m between the house and bat roost	10	(100%)	6	(60%)	16	(80%)
N(%) Household has experienced disturbances from bats	0	(0%)	1	(10%)	1	(5%)
N(%) Reported types of disturbance from bats						
Bat entering the house	0	(0%)	1	(10%)	1	(5%)
Water contamination by bat	0	(0%)	1	(10%)	1	(5%)
Smell of bat urine and guano	0	(0%)	1	(10%)	1	(5%)
Bat excreta	0	(0%)	1	(10%)	1	(5%)
Noise	0	(0%)	1	(10%)	1	(5%)
Fear of disease	0	(0%)	1	(10%)	1	(5%)
Mean (sd) Reported number of bats roosts on the farm	3.5	(1.23)	NR	NR	NR	NR
N(%) Respondent can not count bat in the farm	9	(90%)	NR	NR	NR	NR
N(%) Reported type bat roost						
Constructed on trees	2	(20%)	NR	NR	NR	NR
Constructed on concrete/wooden Poles	8	(80%)	NR	NR	NR	NR

Abbreviation: Bat guano-producing household (BGPH), Non-bat guano-producing household (NBGPH), Standard Deviation (sd), Not relevant (NR)

DETECTION OF CORONAVIRUS IN FOOD, WATER, AND SURFACES

We conducted environmental sampling of food, water, and household surfaces in 10 GBPHs and 10 NGBPHs with the results described in Tables 8 and 9. We detected Infectious Bronchitis Virus (IBV) RNA on 1 (1.4%) of 70 food samples collected from all households (Table 8). IBV was found on a coconut at a BGPH (Table 9). We also detected *Alphacoronavirus* and IBV on 11 (2.9%) of 376 household surface samples (Table 8). Most *Alphacoronavirus*-positive surfaces are bat roosts that were sampled from BGPHs 1, 4, 9, 11, and 14, but an outside table at BGPH 9 also had detectable *Alphacoronavirus* (Table 9). In addition, various surface samples such as food covers at BGPH 12, a kitchen table at NBGPH 10, outside tables at NBGPH 16, and an upstairs table at NBGPH 15 were found positive for IBV (Table 9). No contamination of water samples was detected (Table 8).

TABLE 8. LOCATION AND NUMBER OF FOOD, WATER, AND SURFACE SAMPLES DETECTED POSITIVE FOR *ALPHACORONAVIRUS* AND INFECTIOUS BRONCHITIS VIRUS IN EACH OF 20 BAT AND NON-BAT GUANO-PRODUCING HOUSEHOLDS.

Household ID	Number (%) of positive samples of food, water, and household surfaces			Type of positive samples
	Food	Surface	Water	
Bat guano producing households (BGPH)				
BGPH 1	0 (0.0)	1 (3.8)	0 (0.0)	Bat roost
BGPH 4	0 (0.0)	2 (10.0)	0 (0.0)	Bat roosts
BGPH 6	0 (0.0)	0 (0.0)	0 (0.0)	
BGPH 9	0 (0.0)	2 (11.1)	0 (0.0)	Outside table, bat roosts
BGPH 10	0 (0.0)	0 (0.0)	0 (0.0)	
BGPH 11	1 (25.0)	1 (4.5)	0 (0.0)	Coconut, bat roost
BGPH 12	0 (0.0)	1 (4.5)	0 (0.0)	Food cover
BGPH 14	0 (0.0)	1 (5.0)	0 (0.0)	Bat roost
BGPH 15	0 (0.0)	0 (0.0)	0 (0.0)	
BGPH 16	0 (0.0)	0 (0.0)	0 (0.0)	
Non-bat guano producing households (NBGPH)				
NBGPH 1	0 (0.0)	0 (0.0)	0 (0.0)	
NBGPH 4	0 (0.0)	0 (0.0)	0 (0.0)	
NBGPH 6	0 (0.0)	0 (0.0)	0 (0.0)	
NBGPH 9	0 (0.0)	0 (0.0)	0 (0.0)	
NBGPH 10	0 (0.0)	1 (6.3)	0 (0.0)	Kitchen table
NBGPH 11	0 (0.0)	0 (0.0)	0 (0.0)	
NBGPH 12	0 (0.0)	0 (0.0)	0 (0.0)	
NBGPH 14	0 (0.0)	0 (0.0)	0 (0.0)	
NBGPH 15	0 (0.0)	1 (6.3)	0 (0.0)	Upstairs table
NBGPH 16	0 (0.0)	1 (6.3)	0 (0.0)	Outside table
Total	1 (1.4)	11 (2.9)	0 (0.0)	

TABLE 9. TYPES OF VIRUSES DETECTED IN POSITIVE SURFACE AND FOOD SAMPLES

Virus	Type of positive sample	Household ID	Closest Genbank Match
Alphacoronavirus	Bat roost	BGPH 14	Alphacoronavirus sp. strain VZ_AlphaCoV_16715_47_c2, complete genome (99.7%)
	Bat roost	BGPH 1	Alphacoronavirus sp. strain VZ_AlphaCoV_16715_47_c2, complete genome (99.3%)
	Bat roost	BGPH 4	Alphacoronavirus sp. strain VZ_AlphaCoV_16715_47_c2, complete genome (99.0%))
	Bat roost	BGPH 9	Alphacoronavirus sp. strain VZ_AlphaCoV_16715_47_c2, complete genome (99.7%)
	Outside table	BGPH 9	Alphacoronavirus sp. strain VZ_AlphaCoV_16715_61, complete genome (99.0%)
	Bat roost	BGPH 11	Alphacoronavirus sp. strain VZ_AlphaCoV_16715_63, complete genome (98.3%)
	Bat roost	BGPH 4	Alphacoronavirus sp. strain VZ_AlphaCoV_16715_7, complete genome (100%)
Infectious Bronchitis Virus	Kitchen table	NBGPH 10	Infectious bronchitis virus isolates CK/CH/GD/QY16, complete genome (99.7%)
	Outside table	NBGPH 16	Infectious bronchitis virus strain cK/CH/LSD/110856, complete genome (%)
	Coconut	BGPH 11	Infectious bronchitis virus strain gammaCoV/ck/China/I0347/11, complete genome (%)
	Food cover	BGPH 12	Infectious bronchitis virus strain gammaCoV/ck/China/I0347/11, complete genome (99.7%)
	Upstairs table	NBGPH 15	Infectious bronchitis virus strain gammaCoV/ck/China/I0347/11, complete genome (98.7%)

DISCUSSION

We interviewed 20 households and collected 521 samples of food, water, and household surfaces to understand the contamination level of food, water, and surfaces at households in the bat guano-producing community of Kampong Cham province. We found people's living quarters are very close to bat roosts, and some households in the bat guano-producing community lack water treatment and management, food practices, and hygiene and sanitation practices. The most notable findings were: 1) tap water inside the house is the main water source in most BGPHs and half of NBGPHs, and BGPHs have better water practices (covering storage container of water and treating water at sources and in the house) than NBGPHs, 2) the majority of BGPHs and NBGPHs do not cover their food while drying outside, 3) few households have places for hand washing and 4) household surfaces were detected positive of *Alphacoronavirus* in some BGPHs and NBGPHs. We also found that one food sample (1.4%) and 11 household surface samples (2.9%) were contaminated with either *Alphacoronavirus* or Infectious Bronchitis Virus (IBV) RNA. To our knowledge, this is the first recorded study of household and neighbor survey and sampling on food, water, and household surfaces for viral contamination, which has not been investigated before.

The survey results showed safer water practices in BGPHs than NBGPHs, although most households use tap water as their source of drinking water. The majority (80%) of BGPHs used the tap inside the house for drinking water and had protected containers for water storage, while 50% of NBGPHs had drinking water from the tap in the house, and 80% of NBGPHs covered water storage containers. This suggested that some households in the bat guano-producing community might be using unsafe drinking water. In addition, not all households reported good water treatment, and the households treating water using boiling was higher at BGPHs in comparison to NBGPHs. This result indicates that some BGPHs and most NBGPHs have poor water treatment, and therefore there is concern that their drinking water may not be safe because of their proximity to bat roosts. Furthermore, less than half of NBGPHs and more than half of BGPHs covered storage containers of vegetable gardening water. It is possible that fecal–oral contamination may occur in households that do not cover their containers of vegetable gardening water in the bat guano-producing community. While we did not recover coronavirus RNA from any water samples, such contamination cannot be ruled out based on a single sampling period using small volume grab samples. This study focused primarily on coronavirus spillover risk, but many other pathogens may be contained in bat feces, so better water protection practices among NBGPH may be warranted.

Food safety practices could be improved to reduce the risk of bat contamination for households in the bat guano-producing community. Less than half of the total households surveyed stored food and drinks in fridges, bags, and cool boxes, indicating the need to improve

and encourage appropriate food storage to reduce the risk of contamination. In addition, the majority of BGPHs and NBGPHs dried food or meat without protective covers outside the houses close to the bat roots (less than 20 m), which presents the possibility of contamination from bat feces and urine. Therefore, food safety interventions at households in the bat guano-producing community are needed to reduce the risk of viral spillover from bats to humans.

Hygiene and sanitation practices, especially handwashing, need to be improved in the bat guano-producing community to prevent the spread of viruses from bats. Most households have places for cooking, dish washing and laundry inside the house. All households reported using soap to wash hands, but only two BGPHs have places for hand washing. In addition, the percentage of households using running water to wash hands is low (20%). Almost all households used individual household latrines which might cause an increased risk of poor health outcomes connected to shared sanitation. All of these suggested a lack of hygiene and sanitation that facilitates the spread of disease or virus and is a predicted risk factor for households in the bat guano-producing community.

This study has some limitations. First, participants were not randomly selected for the household survey and sampling of environmental samples since the survey and sampling was conducted depending on their availability and if they would like to participate, and if we could conduct the survey in their homes, though it is worthy of note that more than half of all BGPH (10 of 17 total) were sampled. Second, only limited phylogenetic analysis has been done to date. More in-depth analysis of the sequences will be conducted in the future including the sequence data obtained from the bat guano samples collected from the same farms. Lastly, untreated household water samples were not tested, and so our conclusions regarding water contamination are limited to the direct drinking water sources at households in the bat guano-producing community.

The detection of an *Alphacoronavirus* from a household environmental surface (specifically an outside table at bat guano-producing household) is an interesting finding. This sequence was almost identical to the corresponding region of a whole genome sequence obtained from a *S. kuhlii* guano sample from a guano farm only 200 km south of our study site in Dong Thap province, Viet Nam (Phan et al 2018). On this basis, we consider it highly likely that this viral RNA derives from the bat guano and is possibly the same or a closely related bat-associated coronavirus. A great diversity of *Alphacoronaviruses* has been documented in bats, but to date none have been associated with spillovers to humans, nor are there any presently known to be highly pathogenic in humans, though most bat associated *Alphacoronaviruses* are poorly understood and very few have been isolated in live culture.

While at first glance, detection of Infectious Bronchitis Virus from the surfaces in and around homes may seem relatively insignificant in relation to spillover risk of bat-borne viruses, as it is non-zoonotic and only associated with poultry. Nonetheless, this finding is informative. It

indicates the existence of a pathway for animal-origin viruses to move into homes, onto food preparation surfaces and even onto the surfaces of food. This same virus was also detected in bat guano samples from the same households (reported elsewhere), underlining the close contact of livestock with bat guano. This finding provides a case study in the potential for zoonotic viruses present on guano farms (from any species) to spillover to humans if adequate preventive measures are not in place.

Although coronavirus RNA, including some of likely bat origin, was detected on household surfaces, the exact pathway by which it came to be there is unknown and further study will be necessary to understand the behaviors that result in contaminated food and household surfaces. In addition, future research needs to focus on detailed information about food exposure and hygiene and sanitation practices especially handwashing and surface disinfection to inform more effective community-level risk reduction interventions.

The results from the study will help inform the design of community-level risk reduction interventions through first TIPs and then broader SBC campaigns involving training, counseling and awareness raising at bat-human interface communities. Our results from the survey and laboratory testing suggested that hygiene and contamination interventions of food, water, and surfaces at bat and non-bat guano-producing households in Khchau commune, Kang Meas district, are justified to mitigate the risk of viral spillover from bats to humans with some recommended measures as follows:

- All members of bat and non-bat guano-producing households should wash their hands at critical times, particularly after involvement in activities that are exposed to bat guano.
- All households, especially bat guano-producing households, should have dedicated space for handwashing.
- Both bat and non-bat guano-producing households should frequently clean and disinfect high-touch surfaces and materials that are close to the bat roosts, especially those used for food preparation.
- Families in the bat guano-producing community should properly cover food while drying outside and foods that are stored in open containers in open kitchens.
- Families in the bat guano-producing community should closely cover all water sources outside the home.

These measures are likely to be effective in reducing risk of spillover of coronaviruses from bats to humans in the bat guano-producing community.

CONCLUSIONS

The food, water, and surface assessment identified a knowledge gap for the possibility of viral spillover and transmission; the understanding of the contamination level on food, water, and surfaces at households in the bat guano-producing community; and a need for research for intervention recommendations. Based on the survey results, people living in the bat guano-producing community may be subject to the risk of viral contamination from bats. The proximity of households to bat roosts, the lack of appropriate food and water management, and hygiene practices at households of bat and non-bat guano producers provide opportunities for pathogen spillover. Further research on hygiene and contamination interventions for food, water, and surfaces, particularly handwashing and surface disinfection, is needed to continue developing recommendations and evidence-based interventions for interrupting coronavirus and other virus transmission from bats in the bat guano-producing community. Although uncertainty remains as to whether the species of bats found on bat guano farms harbor viruses pathogenic in humans, the sampling of food, water, and household surfaces showed that surfaces are the primary samples where coronavirus was detected. Food and water samples were less likely to be contaminated with coronaviruses. In addition, IBV was found on various surfaces inside and outside the house, including food surfaces, providing clear evidence of the movement of viral RNA around the premises from animals to the interior of homes and onto food. These findings suggested that a focus on surface disinfection, handwashing, and food covering as priority interventions in bat guano-producing communities could reduce viral spillover risk.

LITERATURE CITED

Bilal, M., Nazir, M. S., Rasheed, T., Parra-Saldivar, R., & Iqbal, H. M. N. (2020). Water matrices as potential source of SARS-CoV-2 transmission – An overview from an environmental perspective. *Case Studies in Chemical and Environmental Engineering*, *2*, 100023. https://doi.org/10.1016/j.cscee.2020.100023

Biryukov, J., Boydston, J. A., Dunning, R. A., Yeager, J. J., Wood, S., Reese, A. L., Ferris, A., Miller, D., Weaver, W., Zeitouni, N. E., Phillips, A., Freeburger, D., Hooper, I., Ratnesar-Shumate, S., Yolitz, J., Krause, M., Williams, G., Dawson, D. G., Herzog, A., … Altamura, L. A. (2020). Increasing Temperature and Relative Humidity Accelerates Inactivation of SARS-CoV-2 on Surfaces. *MSphere*, *5*(4), 10.1128/msphere.00441-20. https://doi.org/10.1128/msphere.00441-20

Bloise, I., Gómez-Arroyo, B., & García-Rodríguez, J. (2020). Detection of SARS-CoV-2 on high-touch surfaces in a clinical microbiology laboratory. *Journal of Hospital Infection*, *105*(4), 784–786. https://doi.org/10.1016/j.jhin.2020.05.017

Ceylan, Z., Meral, R., & Cetinkaya, T. (2020). Relevance of SARS-CoV-2 in food safety and food hygiene: Potential preventive measures, suggestions and nanotechnological approaches. *VirusDisease*, *31*(2), 154–160. https://doi.org/10.1007/s13337-020-00611-0

Chua, K. B. (2003). Nipah virus outbreak in Malaysia. *Journal of Clinical Virology*, *26*(3), 265–275. https://doi.org/10.1016/S1386-6532(02)00268-8

Cui, J., Li, F., & Shi, Z.-L. (2019). Origin and evolution of pathogenic coronaviruses. *Nature Reviews Microbiology*, *17*(3), Article 3. https://doi.org/10.1038/s41579-018-0118-9

Delaune, D., Hul, V., Karlsson, E. A., Hassanin, A., Ou, T. P., Baidaliuk, A., Gámbaro, F., Prot, M., Tu, V. T., Chea, S., Keatts, L., Mazet, J., Johnson, C. K., Buchy, P., Dussart, P., Goldstein, T., Simon-Lorière, E., & Duong, V. (2021). A novel SARS-CoV-2 related coronavirus in bats from Cambodia. *Nature Communications*, *12*(1), 6563. https://doi.org/10.1038/s41467-021-26809-4

Döhla, M., Schulte, B., Wilbring, G., Kümmerer, B. M., Döhla, C., Sib, E., Richter, E., Ottensmeyer, P. F., Haag, A., Engelhart, S., Eis-Hübinger, A. M., Exner, M., Mutters, N. T., Schmithausen, R. M., & Streeck, H. (2022). SARS-CoV-2 in Environmental Samples of Quarantined Households. *Viruses*, *14*(5), 1075. https://doi.org/10.3390/v14051075

Fedorenko, A., Grinberg, M., Orevi, T., & Kashtan, N. (2020). *Virus survival in evaporated saliva microdroplets deposited on inanimate surfaces* (p. 2020.06.15.152983). bioRxiv. https://doi.org/10.1101/2020.06.15.152983

Field, H., Young, P., Yob, J. M., Mills, J., Hall, L., & Mackenzie, J. (2001). The natural history of Hendra and Nipah viruses. *Microbes and Infection*, *3*(4), 307–314. https://doi.org/10.1016/S1286-4579(01)01384-3

Hu, X., Xing, Y., Ni, W., Zhang, F., Lu, S., Wang, Z., Gao, R., & Jiang, F. (2020). Environmental contamination by SARS-CoV-2 of an imported case during incubation period. *Science of The Total Environment*, *742*, 140620. https://doi.org/10.1016/j.scitotenv.2020.140620

Islam, A., Hossain, M. E., Rostal, M. K., Ferdous, J., Islam, A., Hasan, R., Miah, M., Rahman, M.,

Rahman, M. Z., Daszak, P., & Epstein, J. H. (2020). Epidemiology and Molecular Characterization of Rotavirus A in Fruit Bats in Bangladesh. *EcoHealth*, *17*(3), 398–405. https://doi.org/10/gmwcqz

Jiang, F.-C., Jiang, X.-L., Wang, Z.-G., Meng, Z.-H., Shao, S.-F., Anderson, B. D., & Ma, M.-J. (2020). Detection of Severe Acute Respiratory Syndrome Coronavirus 2 RNA on Surfaces in Quarantine Rooms. *Emerging Infectious Diseases*, *26*(9), 2162–2164. https://doi.org/10.3201/eid2609.201435

Kratzel, A., Steiner, S., Todt, D., V'kovski, P., Brueggemann, Y., Steinmann, J., Steinmann, E., Thiel, V., & Pfaender, S. (2020). Temperature-dependent surface stability of SARS-CoV-2. *Journal of Infection*, *81*(3), 452–482. https://doi.org/10.1016/j.jinf.2020.05.074

Le Guyader, F.S., Le Saux, J.C., Ambert-Balay, K., Krol, J., Serais, O., Parnaudeau Giraudon, H., et al. (2008) Aichi virus, norovirus, astrovirus, enterovirus, and rotavirus involved in clinical cases from a French oyster-related gastroenteritis outbreak. J Clin Microbiol. https://doi.org/10.1128/JCM.01044-08.

Lee, S.-E., Lee, D.-Y., Lee, W.-G., Kang, B., Jang, Y. S., Ryu, B., Lee, S., Bahk, H., & Lee, E. (2020). Detection of Novel Coronavirus on the Surface of Environmental Materials Contaminated by COVID-19 Patients in the Republic of Korea. *Osong Public Health and Research Perspectives*, *11*(3), 128–132. https://doi.org/10.24171/j.phrp.2020.11.3.03

Liu, Y., Li, T., Deng, Y., Liu, S., Zhang, D., Li, H., Wang, X., Jia, L., Han, J., Bei, Z., Li, L., & Li, J. (2021). Stability of SARS-CoV-2 on environmental surfaces and in human excreta. *Journal of Hospital Infection*, *107*, 105–107. https://doi.org/10.1016/j.jhin.2020.10.021

Lv, J., Yang, J., Xue, J., Zhu, P., Liu, L., & Li, S. (2020). Detection of SARS-CoV-2 RNA residue on object surfaces in nucleic acid testing laboratory using droplet digital PCR. *Science of The Total Environment*, *742*, 140370. https://doi.org/10.1016/j.scitotenv.2020.140370

Marshall, D. L., Bois, F., Jensen, S. K. S., Linde, S. A., Higby, R., Rémy-McCort, Y., Murray, S., Dieckelman, B., Sudradjat, F., & Martin, G. G. (2020). Sentinel Coronavirus environmental monitoring can contribute to detecting asymptomatic SARS-CoV-2 virus spreaders and can verify effectiveness of workplace COVID-19 controls. *Microbial Risk Analysis*, *16*, 100137. https://doi.org/10.1016/j.mran.2020.100137

Miranda, R. C., & Schaffner, D. W. (2019). Virus risk in the food supply chain. *Current Opinion in Food Science*, *30*, 43–48. https://doi.org/10.1016/j.cofs.2018.12.002

Phan, M. V. T., Ngo Tri, T., Hong Anh, P., Baker, S., Kellam, P., & Cotten, M. (2018). Identification and characterization of Coronaviridae genomes from Vietnamese bats and rats based on conserved protein domains. *Virus Evolution*, *4*(2), vey035. https://doi.org/10.1093/ve/vey035

Purnama, S. G., & Susanna, D. (2020). Hygiene and Sanitation Challenge for COVID-19 Prevention in Indonesia. *Kesmas: Jurnal Kesehatan Masyarakat Nasional (National Public Health Journal)*, *0*, Article 0. https://doi.org/10.21109/kesmas.v15i2.3932

Sattar, S. A., Lloyd-Evans, N., Springthorpe, V. S., & Nair, R. C. (1986). Institutional outbreaks of rotavirus diarrhea: Potential role of fomites and environmental surfaces as vehicles for virus transmission. *Epidemiology & Infection*, *96*(2), 277–289.

https://doi.org/10.1017/S0022172400066055

USAID. (2020). *PREDICT Cambodia: One Health In Action (2009-2020)*. https://static1.squarespace.com/static/5c7d60a711f7845f734d4a73/t/5f24d1a3df814e31ef49c31f/1596248503271/FINAL+REPORT+COUNTRY-CAMBODIA-full.pdf

van Doremalen, N., Bushmaker, T., Morris, D. H., Holbrook, M. G., Gamble, A., Williamson, B. N., Tamin, A., Harcourt, J. L., Thornburg, N. J., Gerber, S. I., Lloyd-Smith, J. O., de Wit, E., & Munster, V. J. (2020). Aerosol and Surface Stability of SARS-CoV-2 as Compared with SARS-CoV-1. *New England Journal of Medicine*, *382*(16), 1564–1567. https://doi.org/10.1056/NEJMc2004973

Velebit, B., Djordjevic, V., Milojevic, L., Babic, M., Grkovic, N., Jankovic, V., & Yushina, Y. (2019). The common foodborne viruses: A review. *IOP Conference Series: Earth and Environmental Science*, *333*(1), 012110. https://doi.org/10.1088/1755-1315/333/1/012110

Wang, L., Su, S., Bi, Y., Wong, G., & Gao, G. F. (2018). Bat-Origin Coronaviruses Expand Their Host Range to Pigs. *Trends in Microbiology*, *26*(6), 466–470. https://doi.org/10.1016/j.tim.2018.03.001

Wong, J. C. C., Hapuarachichi, H. C., Arivalan, S., Tien, W. P., Koo, C., Mailepessov, D., Kong, M., Nazeem, M., Lim, M., & Ng, L. C. (2020). *Environmental Contamination of SARS-CoV-2 in a Non-Healthcare Setting Revealed by Sensitive Nested RT-PCR (preprint)*. http://medrxiv.org/cgi/content/short/2020.05.31.20107862

Yekta, R., Vahid-Dastjerdi, L., Norouzbeigi, S., & Mortazavian, A. M. (2021). Food products as potential carriers of SARS-CoV-2. *Food Control*, *123*, 107754. https://doi.org/10.1016/j.foodcont.2020.107754

APPENDICES

APPENDIX A: SURVEY QUESTIONNAIRE

**HOUSEHOLD SURVEY**						
Thank you for agreeing to participate in the survey. Before getting started, I need to write down my name, the date, time, and location. I will also take GPS coordinates. Please give me a minute.						
**A**	Interviewer					
**B**	Date					
**C**	Time					
**D**	Location					
**PART A: GENERAL INFORMATION**						
**Q1**	Are you the person most responsible for chores like cleaning or cooking in your household?	Yes<br>***[Go to Q3]***	1	No	0	
**Q2**	Is the person most responsible for chores like cleaning or cooking in your household available?	Yes ***[Start new survey with resp. person]***	1	No ***[Continue]***	0	
**Q3**	***[Circle respondent's gender]***	Male	1	Female	0	
**Q4**	What is your name?					
**Q5**	What is your age?		Years			

**HOUSEHOLD SURVEY**							
**Q6**	What is your address?		District		Commune		Village
**Q7**	What is your phone number?						
**Q8**	RECORD GPS location of household		Lon		Lat		Alt
**Q9**	Did you go to school?			Yes	1	No ***[Go to Q11]***	0
**Q10**	What is the highest grade you completed in school?					Grade	
**Q11**	Is the female head of house able to read and write?	Yes	1	No	0	No female HOH	99
**Q12**	Is the male head of the house able to read and write?	Yes	1	No	0	No male HOH	99
**Q13**	***[Observe walls]***	Concrete	1	Plastic, tarp	2	Wood	3
		Dirt	4	Metal	5	Brick	6
		Other: ______________________________					99
**Q14**	***[Observe floor]***	Concrete	1	Dirt	2	Wood	3
		Other: ______________________________					99
**Q15**	***[Observe roof]***	Metal	1	Cloth	2	Plastic, tarp	3
		Wood	4	Grass	5	Other: ______	99

**HOUSEHOLD SURVEY**							
**Q16**	Does your household have wired electricity?			Yes	1	No	0
**Q17**	How many sleeping places do you have in the house?					Beds	
**PART B: DRINKING WATER**							
**Q18**	Where does your household collect drinking water most often?	Protected well	1	Open well	2	Tanker truck	3
		Protected spring	4	Unprotected spring	5	Surface source (river, stream)	6
		Kiosk or tap not at the house	7	Tap at the house	8	Purchased bottle/bag	9
		Rainwater	10	Other: ___________________			99
**Q19**	How many times a day does someone from your household usually collect water?					Times	
						Many times	99
**Q20**	Approximately how long does it take to go collect water and come back?					Minutes	
						Hours	
**Q21**	What other sources do you collect drinking water from? ***[Multiple answers possible]***	Protected well	1	Open well	2	Tanker truck	3
		Protected spring	4	Unprotected spring	5	Surface source (river, stream)	6

HOUSEHOLD SURVEY							
		Kiosk or tap not at the house	7	Tap at the house	8	Purchased bottle/bag	9
		Rainwater	10	Other: ____________________			99
**Q22**	Do you collect water that is not for drinking - for instance for washing dishes or bathing - from a different source?	Yes	1	No ***[Go to Q24]***	0	Don't know ***[Go to Q24]***	99
**Q23**	What sources do you collect non-drinking water from? ***[Multiple answers possible]***	Protected well	1	Open well	2	Tanker truck	3
		Protected spring	4	Unprotected spring	5	Surface source (river, stream)	6
		Kiosk or tap not at the house	7	Tap at the house	8	Purchased bottle/bag	9
		Rainwater	10	Other: ____________________			99
**Q24**	Do you think you can get sick from water?	Yes	1	No ***[Go to Q22]***	0	Don't know ***[Go to Q22]***	99
**Q25**	What kind of sickness can you get from drinking water?	Diarrhea	1	Vomiting	2	Stomach ache	3
		Fever	4	Cholera	5	Dehydration	6

HOUSEHOLD SURVEY								
	[Probe] Any more? ***[Multiple answers possible]***	Headache	7	Influenza	8	General pain	9
		Parasites	10	Other: ___________________			99
**Q26**	How do you know if your water is safe to drink? ***[Probe]***Any other reason? ***[Multiple answers possible]***	Water is clear	1	No bacteria	2	I treat it	3
		Bottled or purchased	4	Used for a long time and not sick	5	I don't think it's safe	6
		Don't know	88	Other: ___________________			99
**Q27**	How might you know if your water is not safe to drink? ***[Probe]***Any other reason? ***[Multiple answers possible]***	Looks dirty	1	Has bacteria	2	Bad source	3
		Makes you sick	4	Not treated	5	Stored in open container	6
		Don't know	88	Other: ___________________			99
**Q28**	Please tell me about any methods you know of for making your water safe to drink. [Circle Yes or No for each] [Probe] Do you know any more ways to make your water safe to drink? Now I would like to know if you have ever used any of those methods to treat your drinking water, and how often.	**Method**		**Known**	Used *[circle one]*		
		Boiling		Y / N	No Rarely Often Always		
		Liquid chlorine		Y / N	No Rarely Often Always		
		Cloth filter		Y / N	No Rarely Often Always		
		Other filter		Y / N	No Rarely Often Always		

HOUSEHOLD SURVEY							
	You said you know about [boiling].<br>Do you use [boiling]?<br>How often do you use [boiling]?	Tablets		Y / N	No Rarely Often Always		
		Other: ___________		Y / N	No Rarely Often Always		
		None					99
**Q29**	Do you believe that the water you have in your house today is safe to drink?			Yes	1	No	0
				Don't know	88	No water	99
**Q30**	May I see the water that you have to drink, or the container where you would store water if you don't have any?			Yes	1	No / refuse ***[Go to Q51]***	0
**Q31**	***[Observe. In what type of container is the water stored?]***	Metal pot	1	Earthen pot	2	Jerrycan	3
		Plastic bottle	4	Bucket	5	Barrel	6
		No container ***[Go to Q36]***	88	Other: ______________________			99
**Q32**	***[Observe. Approximately how many liters is the storage container?]***					Liters	
**Q33**	***[Observe. Is the storage container covered?]***			Yes	1	No	0
**Q34**	***[Observe. Is there water in the container?]***			Yes	1	No ***[Go to Q33]***	0

HOUSEHOLD SURVEY							
**Q35**	Can you please show me how you would get water out of the container? ***[Observe]***			Dips cup into container	1	Pours water out of container	2
				From container's tap	3	Other: ________	99
**Q36**	What source did this water come from?	Protected well	1	Open well	2	Tanker truck	3
		Protected spring	4	Unprotected spring	5	Surface source (river, stream)	6
		Kiosk or tap not at the house	7	Tap at the house	8	Purchased bottle/bag	9
		Rainwater	10	Other: ___________________			99
**Q37**	Can you please give me a cup of water that you would drink? RECORD: PICTURE OF DRINKING WATER STORAGE CONTAINER.			Yes	1	No ***[Go to Q47]***	0
**Q38**	***[Observe. Is that the water shown before (Q30)?]***			Yes ***[Go to Q44]***	1	No	0
**Q39**	***[Observe. In what type of container is the water stored?]***	Metal pot	1	Earthen pot	2	Jerrycan	3
		Plastic bottle	4	Bucket	5	Barrel	6

HOUSEHOLD SURVEY							
		No container ***[Go to Q39]***	88	Other: ______________________			99
**Q40**	***[Observe. Approximately how many liters is the storage container?]***					Liters	
**Q41**	***[Observe. Is the storage container covered?]***			Yes	1	No	0
**Q42**	Can you please show me how you would get water out of the container? ***[Observe]***			Dips cup into container	1	Pours water out of container	2
				From container's tap	3	Other: _____	99
**Q43**	What source did this water come from?	Protected well	1	Open well	2	Tanker truck	3
		Protected spring	4	Unprotected spring	5	Surface source (river, stream)	6
		Kiosk or tap not at the house	7	Tap at the house	8	Purchased bottle/bag	9
		Rainwater	10	Other: ___________________			99
**Q44**	Did someone at the source treat this water in any way?	Yes	1	No ***[Go to Q47]***	0	Don't know	88

HOUSEHOLD SURVEY							
						[Go to Q47]	
**Q45**	How was the water treated at the source?	Liquid chlorine	1	Tablets	2	Cloth filter	3
**Q46**	Approximately how long ago was the water treated at the source?					Minutes	
						Hours	
						Days	
						Don't know	88
**Q47**	Did someone in your household treat this water in any way?	Yes	1	No ***[Go to Q51]***	0	Don't know ***[Go to Q51]***	88
**Q48**	How was the water treated?	Boiled	1	Tablets	2	Cloth filter	3
		Other filter	4	Liquid chlorine	5	Don't know	88
		Other: ______________________					99
**Q49**	Approximately how long ago was the water treated?					Minutes	
						Hours	
						Days	

HOUSEHOLD SURVEY							
						Don't know	88
**Q50**	Can you please give me a cup of water from the same source that has not been treated in any way?	Yes	1	No	0	Don't have	99
**Q51**	Do you have water used for vegetable gardening?	Yes	1	No ***[Go to Q58]***	0	Don't have ***[Go to Q58]***	99
**Q52**	***[Observe. Is that the water shown before (Q30)?]***			Yes ***[Go to Q58]***	1	No	0
**Q53**	***[Observe. In what type of container is the water stored?]***	Metal pot	1	Earthen pot	2	Jerrycan	3
		Plastic bottle	4	Bucket	5	Barrel	6
		No container ***[Go to Q56]***	88	Other: ____________________			99
**Q54**	***[Observe. Approximately how many liters is the storage container?]***					Liters	
**Q55**	***[Observe. Is the storage container covered?]***			Yes	1	No	0
**Q56**	What source did this water come from?	Protected well	1	Open well	2	Tanker truck	3
		Protected spring	4	Unprotect ed spring	5	Surface source (river, stream)	6

HOUSEHOLD SURVEY							
		Kiosk or tap not at the house	7	Tap at the house	8	Purchased bottle/bag	9
		Rainwater	10	Other: ___________________			99
**Q57**	Can you please give me a cup of water you use for vegetable gardening? RECORD: PICTURE OF VEGETABLE GARDEN WATER STORAGE CONTAINER.	Yes	1	No	0	Don't have	99
**PART C: FOOD**							
**Q58**	What do you typically drink during the day? ***[Multiple answers possible]***	Water	1	Soft drinks, soda	2	Tea, coffee	3
		Milk	4	Juice	5	Other: ______	99
**Q59**	I would like you to think for a minute about the food your family has eaten over the past week. Have you eaten *[fresh fruit]*? In general, how often do you eat *[fresh fruit]*?	**Food**		**Eat?**	**How often?** *[circle one]*		
		Fresh fruit		Y / N	No Rarely Often Daily		
		Salad, raw vegetables		Y / N	No Rarely Often Daily		
		Cooked vegetables		Y / N	No Rarely Often Daily		
		Chicken, meat		Y / N	No Rarely Often Daily		
		Raw fish, seafood		Y / N	No Rarely Often Daily		

HOUSEHOLD SURVEY							
		Cooked fish, seafood		Y / N	No Rarely Often Daily		
		Cereals (rice, millet)		Y / N	No Rarely Often Daily		
		Dairy (milk, yogurt)		Y / N	No Rarely Often Daily		
		Other: ___________		Y / N	No Rarely Often Daily		
		None					99
**Q60**	Do you do anything to fresh fruit or vegetables before eating?	Wash	1	Disinfect/bleach	2	Only eat cooked food	3
		Peel	4	Don't know	88	Other: ______	99
**Q61**	For eating, do you and your family usually share a communal pot or do you use individual plates?	Communal pot	1	Individual plates	2	Other: ______	99
**Q62**	Do you use silverware/utensils or do you eat with your hands?	Silverware, utensils	1	Hands	2	Both	3
		Other: ____					99
**Q63**	Have you purchased prepared food over the past week?	Yes	1	No	0	Don't know	88
**Q64**	About how many times have you purchased prepared food over the past week?	Daily or more	1	4-5 times	2	2-3 times	3
		Once	4	Don't know	88		

HOUSEHOLD SURVEY							
**Q65**	Can you please show me where food is stored in your household?	Yes	1	No ***[Go to Q67]***	0	Don't have ***[Go to Q67]***	99
**Q66**	***[Observe. What type of storage is it?] [Observe. Is there raw food (uncooked fish, meat, fresh fruit or vegetables) in each container?]***	**Type of container**		**Have?**		**Store raw food?**	
		Fridge		Y / N		Y/N	
		Clay pot		Y / N		Y/N	
		Bag		Y / N		Y/N	
		Plastic container		Y / N		Y/N	
		Metal container		Y / N		Y/N	
		Other: __________		Y / N		Y/N	
**Q67**	Do you think you can get sick from food?	Yes	1	No ***[Go to Q59]***	0	Don't know ***[Go to Q59]***	88
**Q68**	What kind of sickness can you get from food? ***[Probe]***Any more? ***[Multiple answers possible]***	Diarrhea	1	Vomiting	2	Stomach ache	3
		Fever	4	Cholera	5	Dehydration	6
		Headache	7	Influenza	8	General pain	9
		Parasites	10	Other: ___________________			99

HOUSEHOLD SURVEY								
**Q69**	How do you know if your food is safe to eat? ***[Probe]***Any other reason? ***[Multiple answers possible]***	It is cooked	1	It is fresh	2	Kept cool	3
		I prepare it myself	4	Has no mold, is not rotten	5	Doesn't smell bad	6
		Don't know	88	Other: ___________________			99
**Q70**	How might you know if your food is not safe to eat? ***[Probe]***Any other reason? ***[Multiple answers possible]***	Not cooked enough	1	Not fresh	2	Not kept cool	3
		Not from the household	4	Has mold, is rotten	5	Smells bad	6
		Don't know	88	Other: ___________________			99
**Q71**	What is the longest time that you would store prepared food?	One day or less	1	2-3 days	2	Up to one week	3
		More than a week			4	Don't know	88
**Q72**	Do you believe that the food you have in your house today is safe to eat?			Yes	1	No	0
				Don't know	88	No food	99
**Q73**	Have you ever seen a bat touch your food?	Yes	1	No ***[Go to Q75]***	0	Don't know ***[Go to Q75]***	88
**Q74**	What foods have you seen bats touch?		1		2		3
			4		5		6
		Don't know	88	Other: ___________________			99

HOUSEHOLD SURVEY							
**Q75**	Have you ever seen animal feces in or near food in your house?	Yes	1	No	0	Don't know	88
**Q76**	Have you eaten raw or undercooked meat or organs or blood?	Yes	1	No	0	Don't know	88
**Q77**	Do you or your household members ever dry any meat (beef, pork, fish) outside the house?	Yes	1	No ***[Go to Q82]***	0	Don't know ***[Go to Q82]***	88
**Q78**	What foods or meats do you dry outside the house?	Beef	1	Fish	2	Pork	3
		Don't know	88	Other: ___________________			99
**Q79**	Where do your or your household members dry food or meats?			In front of the house	1	Behind the house	2
				Other: ______________________			99
**Q80**	***[Observe. How far do you or your household members dry food or meats from the bat roost?]***	Between 1-5m	1	Between 5-10m	2	Between 15-20m	3
		Between 25-30m	4	Other: ___________________			99
**Q81**	Have you or your household member cover food and meat while drying?	Yes	1	No	0	Don't know	88
**Q82**	Based on what you have seen of risks in the household, please select foods to be		1		2		3

HOUSEHOLD SURVEY							
	sampled, collect the samples, and write the type of food sampled in the answers to this question. Priority food sampling types are: fruits, vegetables, leftovers stored uncovered that are eaten uncooked. RECORD: Photo of food sampled.		4		5		6
		Don't know	88	Other: ______________________			99
**PART D: HYGIENE AND SANITATION**							
**Q83**	Can you please show me where you cook? ***[Observe location]***	Dedicated space inside the house	1	Space outside the house	2	No dedicated space	3
						Refuse	88
**Q84**	Can you please show me where you wash dishes? ***[Observe location]***	Dedicated space inside the house	1	Space outside the house	2	No dedicated space	3
						Refuse ***[Go to Q69]***	88
**Q85**	How often do you wash dishes?	After every meal	1	Once a day	2	Every 2-3 days	3
		Don't know	88	Don't wash dishes ***[Go to Q87]***			99
**Q86**	Can you please show me what you use to wash dishes? ***[Observe]*** ***[Multiple answers possible]***	Detergent, soap	1	Bleach	2	Sand, earth	3
		Brush	4	Sponge	5	Don't know	88

HOUSEHOLD SURVEY								
		Other: ______________________________						99
**Q87**	How do you typically dry dishes?	With a cloth or towel	1	Let dry inside the home	2	Let dry outside the home	3	
		Don't dry	4	Don't know	88	Other: _____	99	
**Q88**	How often do you clean the floor of your house?	Several times a day	1	Daily	2	Every 2-3 days	3	
		Weekly	4	Don't clean ***[Go to Q71]***	5	Don't know	88	
**Q89**	What do you typically use to clean the floor of your house?	Wet mop with water only	1	Wet mop with detergent	2	Wet mop with bleach	3	
		Dry mop	4	Broom, sweeper	5	Don't know	88	
		Other: ______________________________						99
**Q90**	How often do you typically do laundry?	Daily	1	Every 2-3 days	2	Weekly	3	
		Every 2 weeks or less	4	Don't do laundry ***[Go to Q74]***	5	Don't know	88	
**Q91**	Where do you do laundry?	Dedicated space inside the house	1	Dedicated space outside	2	River	3	

HOUSEHOLD SURVEY							
		Other: ___________________________________					99
Q92	What materials do you typically use to clean clothes?	Detergent, soap	1	Bleach	2	Gravel, stones	3
		Don't know	88	Other: ___________________			99
Q93	Can you please show me where you usually use the bathroom?	Private latrine	1	Shared latrine	2	No latrine ***[Go to Q95]***	99
Q94	Now could you please tell me, for each part of the latrine, how often you clean it and what you use for cleaning?<br><br>For instance, how often do you clean the [floor] of the latrine?<br><br>And what do you use to clean the [floor] of the latrine?<br><br>[If a latrine part is not present, write "N/A"]	***Latrine part***		***Cleaning frequency***		***Cleaning materials***	
		Floor					
		Slab					
		Seat					
		Water storage container					
		Water flushing container					
		Door handle					
		Other: _________					
		Other: _________					
Q95	Can you please show me where you usually wash your hands? ***[Observe location]***	Dedicated space	1	No dedicated space	0	Refuse	88
Q96	Can you please show me what you use to wash your	Soap present	1	Soap absent	0	Refuse	88

HOUSEHOLD SURVEY							
	hands?<br>***[Observe if soap is present]***	Other: ______________________					99
**Q97**	***[Observe if water is present]***	Running water	1	Water in container	2	No water	0
**Q98**	Do you have trash in your household (from cooking, for instance)?	Yes	1	No	0	Don't know	88
**Q99**	Can you please show me where you store trash?<br>***[Observe]***	Close/covere d container	1	Bag	2	Dump site close to the house	3
		Other: ______________________					99
**Q100**	What do you do with your trash?	Put on the street for collection	1	Take to dumpsite	2	Burn	3
		Bury	4	Don't know	88	Other: ______	99
**Q101**	How often do you do that action with your trash?	Daily	1	Every 2-3 days	2	Weekly	3
		Every 2 weeks or less			4	Don't know	88
**Q102**	Please draw a scheatic map on the enclosed page of the household. Based on what you have seen of risks in the household, please select surfaces to be sampled, collect the samples, and write the type of surface sampled in		1		2		3
			4		5		6

HOUSEHOLD SURVEY							
	the answers to this question and on the map. Priority surface sampling types are: drying clothes, kitchen implements, water tank covers. RECORD: Picture of surfaces.	Don't know	88	Other: ______________________			99
**PART E: BATS**							
**Q103**	Are there any bats living in your house (in the ceiling or wall cracks)?	Yes	1	No ***[Go to Q105]***	0	Don't know ***[Go to Q105]***	88
**Q104**	How long have the bats lived in your house?	Less than one year	1	1-2 years	2	3-4 years	3
		5-6 years	4	6-7 years	5	More than 8 years up	6
		Other: ______________________					99
**Q105**	How far is your house from the bat roost?	Between 1-20m	1	Between 21-40m	2	Between 41-60m	3
		Between 61-80m	4	Between 81-100m	5	More than 100m	6
		Other: ______________________					99
**Q106**	Have you or your household members experienced any disturbances from the bats?	Yes	1	No ***[Go to Q109]***	0	Don't know ***[Go to Q109]***	88

HOUSEHOLD SURVEY							
**Q107**	If yes, what kind of disturbances have you or your household members experienced? Multiple answers possible.	Bat entered the house	1	Water contamination by bat	2	Smell of bat urine or guano	3
		Bat Excreta	4	Noise	5	Extra cleaning	6
		Fear of disease spread	7	Other: ___________________			99
**Q108**	How many bat roosts are there in the farms?					bat roosts	
**Q109**	Approximately, how many bats are there in your farm?					bat	
**Q110**	What type of each bat roost? Multiple answers possible.	Constructed on trees	1	Constructed on concrete/ wooden poles	2	Cave	3
		Building	4	Other: ___________________			99
**Q111**	***[Indicate collected samples]***	Treated water		Yes	1	No	0
		Untreated water		Yes	1	No	0
		Food Samples		Yes	1	No	0
		Surfaces Samples		Yes	1	No	0
**Q112**	***[Report end time]***			End time			

APPENDIX B: SURVEY CONSENT INFORMATION SHEET

You are being invited to take part in a research study being conducted by Dr. Hellen Amuguni, colleagues from Tufts University and Dr. Pheng Vutha with the Cambodia STOP Spillover country team because you are a household in a community where there is bat guano-production.

If you decide to be in the study, we will ask you questions about how you handle food and water in your house. We are interested in knowing if they could be contaminated by animals such as bats. We will also take swabs and collect a sample of some of your food, your water, and surfaces in the house. We will use those to see if there has been any contamination by animals such as bats. This should take less than two hours. The swabs will be taken to the Institute Pasteur laboratory to test for contamination by animals such as bats. You can eat the food we have swabbed after washing it in water. Swabbing your food or the household surfaces should not damage them in any way. However, if you would like, we either replace your food or give you money to replace the food. Please note we will return to your house to tell you the results of the testing.

Your participation in this study is completely voluntary. You can skip questions that you do not want to answer or stop participating at any time with no penalty to you. The alternative to participating in this research is not to participate.

There is a risk of loss of confidentiality, meaning your private information could be seen by someone outside of the research team. No identifying information about you will be included in any report or publication. The identifying information we collect from you will be disconnected from the study data as soon as possible after we have provided you with the results of the testing. However, we will keep your identifying information in a separate registry so we can contact you in case you might be interested in future related studies. Additionally, we are not going to take any photographs of any people in your home. We would like to take photographs of objects in your home—food, your water storage container, and food, water, and surfaces—without any humans in the pictures.

There are no direct benefits to you from taking part in this study. We cannot promise any benefits to others from your taking part in this study. However, there would be possible benefits to the people living in your communities and country if it helps to reduce the chance that a disease could go from animals such as bats to people.

If you have questions, concerns, or complaints, or think the research has hurt you, please contact Dr. Pheng Vutha, who is the Cambodia Country Team Lead. He is available at vutha.pheng@tetratech.com and by phone at +855 12 697 487. You may also contact Dr. Hellen Amuguni, Principal Investigator, at janetrix.amuguni@tufts.edu.

If you have questions about your rights as a research study subject, you may contact the National Ethics Committee at the Ministry of Health at (855-23) 885-970/884 909. You may also contact the Tufts University Health Sciences Institutional Review Board (IRB) at (617) 636-7512. This study has been reviewed by the Tufts Health Sciences IRB.

APPENDIX C: PARTICIPANT LIST FOR FIELD SURVEY/ SAMPLING

No	Name	Institution
1	Jonathon Gass	STOP Spillover, Tufts University
2	Daniele Lantagne	STOP Spillover, Tufts University
3	Pheng Vutha	STOP Spillover Cambodia
4	Sry Chanty	STOP Spillover Cambodia
5	Neang Sarin	STOP Spillover Cambodia
6	Bun Chan	National Animal Health and Production Research Institute (NAHPRI), General Directorate of Animal Health and Production (GDAHP)
7	Tich Phearun	Provincial Department of Agriculture (PDA)
8	Po Lyma	Provincial Health Department (PHD)
9	Koeng Korsorl Mony	Provincial Department of Environment (PDE)

APPENDIX D: FIELD PROGRAM OF SURVEY AND SAMPLING

Date	Time	Activity	Participant	Note
Apr 24 (Mon)	7:30 - 10:00	Depart for the bat guano producing community in Kampong Cham province	Members from Phnom Penh	
	10:00 - 12:00	Provide training and orientation for the sampling teams and enumerators	All members	• Short ethics briefing to comply with Tufts IRB requirements<br>• Survey tools and sampling methods<br>• Required personal and protective equipment (PPE) and safety measures
	12:00 - 14:00	Lunch and travel to bat guano-producing community	All members	
	14:00 - 17:00	Following the training, conduct household survey and sample collection with all members	All members	• 1 bat guano-producing household<br>• 1 non-bat guano-producing household<br>• 10-15 samples/household
	17:00 ~	Return to Kampong Cham city center		Hotel in Kampong Cham province
Apr 25 (Tue)	8:00 - 9:00	Depart for the bat guano-producing community	All members	
	09:00 - 12:00	Conduct household survey and collect food, water, and surface samples	2 teams	• 2 bat guano-producing households<br>• 2 non-bat guano-producing households<br>• 10-15 samples/household
	12:00 - 13:30	Lunch	All members	
	13:30 - 15:00	Conduct household survey and collect food, water, and surface samples	2 teams	• 2 bat guano-producing households<br>• 2 non-bat guano-producing households<br>• 10-15 samples/household
	17:00 ~	Return to Kampong Cham city center	All members	Hotel in Kampong Cham province
Apr 26 (Wed)	8:00 - 9:00	Depart for the bat guano producing community	All members	

Date	Time	Activity	Participant	Note
	09:00 - 12:00	Conduct household survey and collect food, water, and surface samples	2 teams	• 2 bat guano-producing households<br>• 2 non-bat guano-producing households<br>• 10-15 samples/household
	12:00 - 13:30	Lunch	All members	
	13:30 - 17:00	Conduct household survey and collect food, water, and surface samples	2 teams	• 2 bat guano-producing households<br>• 2 non-bat guano-producing households<br>• 10-15 samples/household
	17:00 ~	Return to Kampong Cham city center	All members	Hotel in Kampong Cham province
27-Apr (Thu)	8:00 - 9:00	Depart to the bat guano-producing community	All members	
	09:00 - 12:00	Conduct household survey and collect food, water, and surface samples	2 teams	• 1 bat guano-producing household<br>• 1 non-bat guano-producing household<br>• 10-15 samples/household
	12:00 - 13:30	Lunch		
	13:30 - 15:00	Conduct household survey and collect food, water, and surface samples	2 teams	Continue survey and sample collection if needed
	17:00 ~	Return to Kampong Cham city center	All members	Hotel at Kampong Cham province
28-Apr (Fri)	8:30 ~	Return to Phnom Penh	Members from Phnom Penh	

APPENDIX E: LIST OF SURVEYED HOUSEHOLDS WITH SITE ID

No	Site ID of bat guano- producing households	Site ID of non-bat guano-producing households	Location		
			Village	District	Province
1	BGPH 1	NBGPH 1	Varint 1	Kang Meas	Kampong Cham
2	BGPH 4	NBGPH 4	Varint 1	Kang Meas	Kampong Cham
3	BGPH 6	NBGPH 6	Varint 1	Kang Meas	Kampong Cham
4	BGPH 9	NBGPH 9	Varint 3	Kang Meas	Kampong Cham
5	BGPH 10	NBGPH 10	Varint 3	Kang Meas	Kampong Cham
6	BGPH 11	NBGPH 11	Varint 3	Kang Meas	Kampong Cham
7	BGPH 12	NBGPH 12	Varint 3	Kang Meas	Kampong Cham
8	BGPH 14	NBGPH 14	Varint 2	Kang Meas	Kampong Cham
9	BGPH 15	NBGPH 15	Varint 2	Kang Meas	Kampong Cham
10	BGPH 16	NBGPH 16	Varint 2	Kang Meas	Kampong Cham
