# Supplementary material for: Community Participatory Approach to Design, Test, and Implement Interventions That Reduce Risk of Bat-Borne Disease Spillover: A Case Study from Cambodia
Source: Trop Med Infect Dis. 2025 Dec 27;11(1):7. doi: 10.3390/tropicalmed11010007 (PMC12846364; doi:10.3390/tropicalmed11010007)
Supplement: Supplementary file 1 [file tropicalmed-11-00007-s001.zip › File S3. Non-biological sampling in bat guano farms.pdf]

File S3: Non-biological sampling in bat guano farms

Table S1. Location and number of food, water and surface samples detected positive of *Alphacoronavirus* and Infectious Bronchitis Virus in each of 20 bat and non-bat guano producing households.

Household ID	Number (%) of positive samples of food, water and household surfaces						Type of positive samples
	Food		Surface		Water		
Bat guano producing households (BGPH)							
BGPH 1	0	(0.0)	1	(3.8)	0	(0.0)	Bat roost
BGPH 4	0	(0.0)	2	(10.0)	0	(0.0)	Bat roosts
BGPH 6	0	(0.0)	0	(0.0)	0	(0.0)	
BGPH 9	0	(0.0)	2	(11.1)	0	(0.0)	Outside table, bat roosts
BGPH 10	0	(0.0)	0	(0.0)	0	(0.0)	
BGPH 11	1	(25.0)	1	(4.5)	0	(0.0)	Coconut, bat roost
BGPH 12	0	(0.0)	1	(4.5)	0	(0.0)	Food cover
BGPH 14	0	(0.0)	1	(5.0)	0	(0.0)	Bat roost
BGPH 15	0	(0.0)	0	(0.0)	0	(0.0)	
BGPH 16	0	(0.0)	0	(0.0)	0	(0.0)	
Non-bat guano producing households (NBGPH)							
NBGPH 1	0	(0.0)	0	(0.0)	0	(0.0)	
NBGPH 4	0	(0.0)	0	(0.0)	0	(0.0)	
NBGPH 6	0	(0.0)	0	(0.0)	0	(0.0)	
NBGPH 9	0	(0.0)	0	(0.0)	0	(0.0)	
NBGPH 10	0	(0.0)	1	(6.3)	0	(0.0)	Kitchen table
NBGPH 11	0	(0.0)	0	(0.0)	0	(0.0)	
NBGPH 12	0	(0.0)	0	(0.0)	0	(0.0)	
NBGPH 14	0	(0.0)	0	(0.0)	0	(0.0)	
NBGPH 15	0	(0.0)	1	(6.3)	0	(0.0)	Upstairs table
NBGPH 16	0	(0.0)	1	(6.3)	0	(0.0)	Outside table
Total	1	(1.4)	11	(2.9)	0	(0.0)	

Table S2. Types of viruses detected in positive surface and food samples.

Virus	Type of positive sample	Household ID	Closest Genbank Match
Alphacoronavirus	Bat roost	BGPH 14	Alphacoronavirus sp. strain VZ_AlphaCoV_16715_47_c2, complete genome (99.7%)
	Bat roost	BGPH 1	Alphacoronavirus sp. strain VZ_AlphaCoV_16715_47_c2, complete genome (99.3%)
	Bat roost	BGPH 4	Alphacoronavirus sp. strain VZ_AlphaCoV_16715_47_c2, complete genome (99.0%))
	Bat roost	BGPH 9	Alphacoronavirus sp. strain VZ_AlphaCoV_16715_47_c2, complete genome (99.7%)
	Outside table	BGPH 9	Alphacoronavirus sp. strain VZ_AlphaCoV_16715_61, complete genome (99.0%)
	Bat roost	BGPH 11	Alphacoronavirus sp. strain VZ_AlphaCoV_16715_63, complete genome (98.3%)
	Bat roost	BGPH 4	Alphacoronavirus sp. strain VZ_AlphaCoV_16715_7, complete genome (100%)
Infectious Bronchitis Virus	Kitchen table	NBGPH 10	Infectious bronchitis virus isolate CK/CH/GD/QY16, complete genome (99.7%)
	Outside table	NBGPH 16	Infectious bronchitis virus strain cK/CH/LSD/110856, complete genome (%)
	Coconut	BGPH 11	Infectious bronchitis virus strain gammaCoV/ck/China/I0347/11, complete genome (%)
	Food cover	BGPH 12	Infectious bronchitis virus strain gammaCoV/ck/China/I0347/11, complete genome (99.7%)
	Upstairs table	NBGPH 15	Infectious bronchitis virus strain gammaCoV/ck/China/I0347/11, complete genome (98.7%)
