# Supplementary material for: Community Participatory Approach to Design, Test, and Implement Interventions That Reduce Risk of Bat-Borne Disease Spillover: A Case Study from Cambodia
Source: Trop Med Infect Dis. 2025 Dec 27;11(1):7. doi: 10.3390/tropicalmed11010007 (PMC12846364; doi:10.3390/tropicalmed11010007)
Supplement: Supplementary file 1 [file tropicalmed-11-00007-s001.zip › File S4. Trials implemented by BGPs and NBGPs during TIPs.pdf]

File S4: Trials implemented by BGPs and NBGPs during TIPs.

Table S1: Trial of PPE consistent use among households.

**Sub-behaviors / Elements** **(Total trials = 11)**	**No. of Initial Practices**	**No. of Agree to Try**	**No. of Succeed**	**No. Intend to Continue**
Head covering	11	11	11	11 HHs
Eye shielding (glasses)	1	11	7	
Mask--Nose/mouth	8	11	10	
Gloves--Hand	4	11	7	
Garment--Upper body	8	11	11	
Garment--Lower body	8	11	10	
Boots--Feet	1	11	8	

Table S2: Trial of PPE storage among households.

**Sub-behaviors** **(Total trials = 11)**	**No. of Initial Practices**	**No. of Agree**	**No. of Succeed**	**No. Intend to Continue**
Take off PPE correctly by removing the gloves last	0	11	4	11 HHs
Allocate a space outside the house (near guano harvest area if possible) for changing and storing PPE.	2	11	10	
Dedicated space with hooks/hangers.	0	11	11	
Dedicated space for washing	0	11	8	
Disinfect all reusable PPE after every use (water, soap, water bucket…)	0	11	8	
Use disinfectant liquid	0	11	3	

Place to dry PPE (free of bats)	0	11	11	
Single use of mask / used correctly	0	11	9	

Table S3: Trial of optimal handwashing among households.

**Sub-behaviors / elements (Total trial= 8)**	**No. Initial Practices**	**No. Agree**	**No. Succeed**	**No. Intend to Continue**
Handwashing station near bat roosts[1]	0	8	1	8 HHs
Handwashing station outside the house	3	5	8	
Presence of water	6	8	8	
Presence of soap	4	8	8	
Presence of clean cloth to dry hands	0	8	5	

Table S4: Trial of optimal guano storage behavior.

**Sub-behaviors (Total trials = 2)**	**No. Initial Practices**	**No. Agree**	**No. Succeed**	**No. Intend to Continue**
Store the guano in two layer-bag	0	2	1	2
Keep guano storage in safe location outside the house, without accessible by domestic animal	0	2	1	2

Table S5: Trial of Cleaning high-touch surfaces daily.

**Sub-behaviors (Total trials = 11)**	**No. Initial Practices**	**No. Agree**	**No. Succeed**	**No. Intend to Continue**

All surfaces in the kitchen or where food is eaten or stored are cleaned with soap and water	*5*	*9*	*9*	11 HHs
Wipe daily in the morning	*1*	*9*	*7*	
Wash the cloth used to wipe the surfaces clothes after cleaning	*0*	*9*	*7*	

Table S6: Trial of protecting exposed food from bat droppings.

**Sub-behaviors**<br>**(Total trials = 4)**	**No. Initial Practices**	**No. Agree**	**No. Succeed**	**No. Intend to Continue**
Food put out to dry is placed as far as possible from the roost.	4	4	4	4 HHs
Food is carefully put out to dry to be sure it is after bats are in roost and food is taken in before the bats leave the roost.	4	4	4	
Food stored in the kitchen or house covered, placed in a bag, jar, box or similar.	1	4	4	

Table S7: Trial of proper disposal of dead bats.

**Sub-behaviors**<br>**(Total trials = 9)**	**No. Initial Practices**	**No. Agree**	**No. Succeed**	**No. Intend to Continue**
Dead bat is immediately disposed of to keep from animals and children	3	*9*	*1*	9 HHs
Put on mask and gloves	1	*9*	*1*	
Pick up the bat with a plastic bag on hand that is pulled down to double cover the bat. Put in another plastic bag.	2	*9*	*1*	
Burn or deeply bury the bat away from home.	2	*9*	*1*	

Remove and clean PPE	**0**	***9***	***1***	
